# Behavior Change Resources Used in Mobile App–Based Interventions Addressing Weight, Behavioral, and Metabolic Outcomes in Adults With Overweight and Obesity: Systematic Review and Meta-Analysis of Randomized Controlled Trials

**DOI:** 10.2196/63313

**Published:** 2025-08-19

**Authors:** Sijia Li, You Zhou, Ying Tang, Haoming Ma, Yuying Zhang, Aoqi Wang, Xingyi Tang, Runyuan Pei, Meihua Piao

**Affiliations:** 1 Chinese Academy of Medical Sciences, Peking Union Medical College School of Nursing, No 33 Ba Da Chu Road, Shijingshan District, Beijing, 100144, China, 86 13522112889; 2 School of Nursing, Evidence-Based Nursing Center, Lanzhou University, Lanzhou, 730011, China

**Keywords:** mobile app, overweight, obesity, mHealth, eHealth, physical activity, diet, app, application, behavior change, behavior, exercise, systematic review, review, meta-analysis, weight, BMI, body fat, systolic blood pressure, diastolic blood pressure, blood pressure, metabolic, obese adults

## Abstract

**Background:**

Overweight and obesity have become a public health issue. Lifestyle modifications delivered through mobile devices, especially mobile phones, present an opportunity to support weight loss efforts. However, evidence regarding the effects of mobile apps on other outcomes, such as blood pressure and physical activity (PA), remains limited. Recent studies on this topic require a systematic review and updating, and the active elements that promote behavior change remain unclear.

**Objective:**

The meta-analysis aimed to explore the effects of mobile phone apps on weight-related outcomes (weight, BMI, waist circumference [WC], fat mass, fat mass percentage), behavioral outcomes (moderate-to-vigorous physical activity [MVPA], energy intake), and metabolic outcomes (systolic blood pressure [SBP], diastolic blood pressure [DBP], triglycerides, hemoglobin A_1c_ [HbA_1c_]) among adults with overweight and obesity. Behavior change techniques (BCTs), the smallest replicable intervention elements, were also identified to clarify the components used in current studies, along with associated resources, including facilitating, boosting, and nudging. In addition, factors influencing the effectiveness of these interventions were explored.

**Methods:**

Six databases (PubMed, Embase, CENTRAL, Web of Science, PsycINFO, and CINAHL) were searched for relevant randomized controlled trials (RCTs) published in English from inception to May 20, 2024. Two independent authors conducted study selection, data extraction, and quality assessment. The effect size of interventions was calculated using the mean difference (MD), and a random-effects model was applied for data analysis. Subgroup and sensitivity analyses were conducted to explore potential influencing factors and identify possible sources of heterogeneity.

**Results:**

A total of 29 studies were included. The results indicated that mobile phone app interventions significantly reduced weight (MD=−1.45 kg, 95% CI −2.01 to −0.89; *P*<.001), BMI (MD=−0.35 kg/m^2^, 95% CI −0.57 to −0.13; *P*=.002), WC (MD=−1.98 cm, 95% CI −3.42 to −0.55; *P*=.007), fat mass (MD=−1.32 kg, 95% CI −1.94 to −0.69; *P*<.001), DBP (MD=−1.76 mm Hg, 95% CI −3.47 to −0.04; *P*=.04), and HbA_1c_ (MD=−0.13%, 95% CI −0.22 to −0.04; *P*=.005). However, nonsignificant effects were observed for other outcomes. The most frequently used BCTs included 2.3 “self-monitoring of behavior” (n=25), 4.1 “instruction on how to perform the behavior” (n=24), 2.2 “feedback on behavior” (n=20), 1.1 “goal setting (behavior)” (n=19), and 1.4 “action planning” (n=15). Fifty-nine percent of included studies used 3 resource types (ie, facilitating, boosting, and nudging). Subgroup analyses identified combined diet and PA interventions, medium-term intervention duration, and the use of ≥8 BCTs as potential reference interventions for improving outcomes.

**Conclusions:**

This meta-analysis demonstrates that mobile phone app interventions significantly reduce weight, BMI, WC, fat mass, DBP, and HbA_1c_ in adults with overweight and obesity. However, future studies should explore ways to optimize app interventions by incorporating behavior change strategies and resources to further enhance their overall effectiveness.

## Introduction

Overweight and obesity are defined as excessive fat accumulation that can negatively affect health. People with a BMI between 25 and 30 kg/m^2^ are classified as overweight, while those with a BMI over 30 kg/m^2^ are classified as obese [1]. Overweight and obesity have become major public health issues. In 2016, more than 1.9 billion adults were overweight, with approximately 650 million experiencing obesity [2], and this number is estimated to affect half of the global population by 2030 [3]. Obesity is associated with an increased incidence of chronic conditions such as type 2 diabetes and hypertension [2], which in turn reduces disease-free years [4], quality of life [5], and life expectancy [6]. In addition, obesity imposes a substantial economic burden on nations [7]. In 2014, the global economic impact of obesity was projected to be US $2.0 trillion, equivalent to 2.8% of the global gross domestic product (GDP) [3]. Compared with the healthy-weight population, individuals with obesity incur 36% higher average annual health care expenses, including 105% higher prescription costs and 39% higher primary care costs [8]. Given the health consequences and economic burden associated with obesity, effective interventions are of great importance.

The fundamental approach to addressing overweight and obesity remains a multicomponent behavioral intervention [9]. However, several barriers exist in implementing lifestyle interventions aimed at weight loss [10]. A previous systematic review [11] has suggested that mobile health (mHealth) apps hold promise for health behavior change. mHealth is defined as “medical and public health practice supported by mobile devices, such as mobile phones, patient monitoring devices, personal digital assistants, and other wireless devices” [12]. In addition, with advancements in technology, the number of mobile phone users has been steadily increasing, and mHealth interventions have the potential to engage large populations at relatively low costs, enhancing the feasibility and accessibility of public health interventions [13]. Therefore, lifestyle modifications delivered via mobile devices, especially mobile phones, present an opportunity to help people lose weight.

Several meta-analyses have evaluated the effectiveness of mobile phone apps on weight loss in adults [14-16]. However, these reviews have some limitations. First, most reviews [14-18] have primarily focused on weight and BMI, while other important outcomes, such as fat mass, blood pressure, and physical activity (PA), have also been reported in original studies. These additional outcomes hold clinical significance and should be included to provide a more comprehensive understanding of the overall effectiveness of mobile app–based interventions. This would offer valuable insights for helping adults with overweight and obesity prevent obesity-related comorbidities. Second, with the rapid development of technology in recent years, numerous new randomized controlled trials (RCTs) have emerged [19-23], necessitating updated evidence. Third, although behavioral interventions are the cornerstone of weight loss, the specific components used in these interventions have not been fully examined from the perspective of behavior change [15,16].

Behavior change techniques (BCTs) are the smallest replicable intervention elements designed to modify or redirect the causal mechanisms that regulate behavior [24,25]. BCTs are widely used to guide and understand intervention design, and identifying the resources associated with these BCTs is crucial for understanding their functional mechanisms [25]. To address this, Michaelsen et al [26] proposed a behavior change resource model (BCRM) centered on an individual’s resources to elucidate the resources used to form these BCTs. This model categorizes BCTs into 3 types: facilitating, boosting, and nudging, which correspond to external resource provision, reflective resource build-up, and affective resource use, respectively. To the best of our knowledge, no review has yet identified the resources used in mobile app–based behavior change interventions.

Therefore, we aimed to conduct a systematic review and meta-analysis to explore the effects of mobile phone apps on weight-related outcomes (weight, BMI, waist circumference [WC], fat mass, and fat mass percentage), behavioral outcomes (moderate-to-vigorous physical activity [MVPA] and energy intake), and metabolic outcomes (systolic blood pressure [SBP], diastolic blood pressure [DBP], triglycerides, and hemoglobin A_1c_ [HbA_1c_]) among adults with overweight and obesity. In addition, we identified the BCTs and behavior change resources (BCRs) used in these interventions. We also explored factors influencing the effectiveness of these interventions.

## Methods

### Overview

This meta-analysis was conducted according to the Preferred Reporting Items for Systematic Reviews and Meta-Analyses (PRISMA) guidelines [27] (the PRISMA checklist is provided in Checklist 1) and was registered on the International Prospective Register of Systematic Reviews (PROSPERO registration number: CRD42024513381).

### Search Strategy

Six databases (ie, PubMed, Embase, CENTRAL, Web of Science, PsycINFO, and CINAHL) were independently searched by 2 authors (SJL and YZ) for relevant RCTs published in English from inception to May 20, 2024. The search strategy was developed by one author (SJL) and confirmed by another author (YZ) according to the PICOS (Participants, Interventions, Comparisons, Outcomes, and Study Design) framework. The key search terms and MeSH (Medical Subject Headings) included obesity, overweight, body weight, mobile apps, mHealth, and apps. The full search strategy is provided in Multimedia Appendix 1. In addition, we manually searched the references of previously published relevant reviews.

### Study Eligibility Criteria

Studies included in this meta-analysis had to meet the following criteria: (1) participants: adults with overweight and obesity aged ≥18 years; (2) intervention: mobile phone apps used as the primary component; (3) comparisons: usual care without mobile phone app interventions or no intervention; (4) outcomes: weight-related outcomes (ie, weight, BMI, WC, fat mass, and fat mass percentage), behavioral outcomes (ie, MVPA and energy intake), and metabolic outcomes (ie, SBP, DBP, triglycerides, and HbA_1c_); (5) study design: RCTs published in English.

Studies were excluded if they met the following criteria: (1) participants diagnosed with diseases other than obesity; (2) conference articles, letters, reviews, commentaries, or protocols; and (3) unavailable full texts or incomplete relevant data.

### Study Selection and Data Extraction

All titles and abstracts of the studies were downloaded and imported into EndNote X9, with duplicates automatically removed. Two investigators (SJL and YZ) independently screened the titles and abstracts based on the eligibility criteria. Subsequently, the full texts of potentially relevant studies were downloaded and reviewed to select the included articles. Reasons for exclusion were recorded. Any disagreements were resolved through consultation with a third author (HMM).

Data regarding paper characteristics (author, year, and country), study design, study population (eg, average age, sex distribution, and baseline BMI), intervention content (eg, intervention duration and brief description of the intervention), comparison content (eg, brief introduction to the comparison), and outcomes were extracted into a predesigned Microsoft Excel by one reviewer (SJL) and checked by a second reviewer (YZ). We did not make any assumptions about missing or unclear information when extracting data to avoid introducing misleading information. Other statistics (eg, 95% CI or SEs) were converted to SD if not available (Cochrane Handbook version 6.4, Chapter 5, 5.7) [28]. If the units of the outcome measures were not uniform, such as triglycerides measured in mg/dL, we converted them to mmol/L to ensure consistency in units. We used Michie BCT Taxonomy (BCTTv1) to identify the BCTs present in the included studies (Multimedia Appendix 2) [24]. Intervention and control descriptors in the original RCTs that aligned with BCT definitions were marked with a “✓.” The initial identification of BCTs was performed by 1 reviewer (SJL) and checked and confirmed by another reviewer (YT). Subsequently, we mapped the BCTs used in each study to the corresponding BCRs.

### Quality Assessment

Cochrane risk-of-bias version 2 (ROB 2) tool was used to assess the methodological quality of the included RCTs by 2 reviewers (SJL and YZ) independently [29]. This tool contains 5 domains: the randomization process, deviations from the intended interventions, missing outcome data, measurement of the outcome, and selection of the reported result. Each domain was rated as either low, some concerns, or high risk of bias. An overall judgment was then assigned to each study. A third investigator (HMM) was responsible for resolving any discrepancies during the evaluation process.

Two independent authors (SJL and YZ) used the Grading of Recommendations, Assessment, Development, and Evaluation (GRADE) framework to assess the overall quality of evidence. The framework evaluates 5 domains: risk of bias, inconsistency, indirectness, imprecision, and publication bias. Based on these evaluations, the quality of evidence was classified as high, moderate, low, or very low. Any disagreements during this process were resolved by a third reviewer (HMM).

### Statistical Analysis

The meta-analysis was performed to evaluate the effectiveness of mobile app interventions on weight loss among adults with overweight and obesity, with forest plots used to visually display the results of individual studies and the overall effect size. Outcomes were pooled in the meta-analysis if change scores from baseline were available, and the number of participants was recorded. The heterogeneity of the data was assessed using the chi-square test and the *I^2^* statistic, with 25%, 50%, and 75% representing low, moderate, and high heterogeneity, respectively [30]. Review Manager software (version 5.3; Cochrane Collaboration) was used to analyze data. The mean difference (MD) with a 95% CI was used to express the effectiveness of interventions involving mobile apps. A random-effects model was applied for data analysis to obtain more conservative results.

Subgroup analyses were conducted to explore factors, including intervention contents, presence of theory, intervention duration, number of BCTs, and number of BCRs, which may influence the effects of the intervention on weight and help identify possible sources of heterogeneity. Sensitivity analyses were also performed to assess the robustness of the results in R (version 4.4.2; R Foundation for Statistical Computing). Publication bias was evaluated by visual evaluation of funnel plot asymmetry and quantified by Egger test in Stata software (version 15; StataCorp). Publication bias assessment was performed separately for outcome measures included in meta-analyses that incorporated more than 10 original studies (Cochrane Handbook version 6.4, Chapter 13, 13.3) [28]. In all analyses, *P*<.05 was considered statistically significant.

## Results

### Study Selection

**Figure 1.**
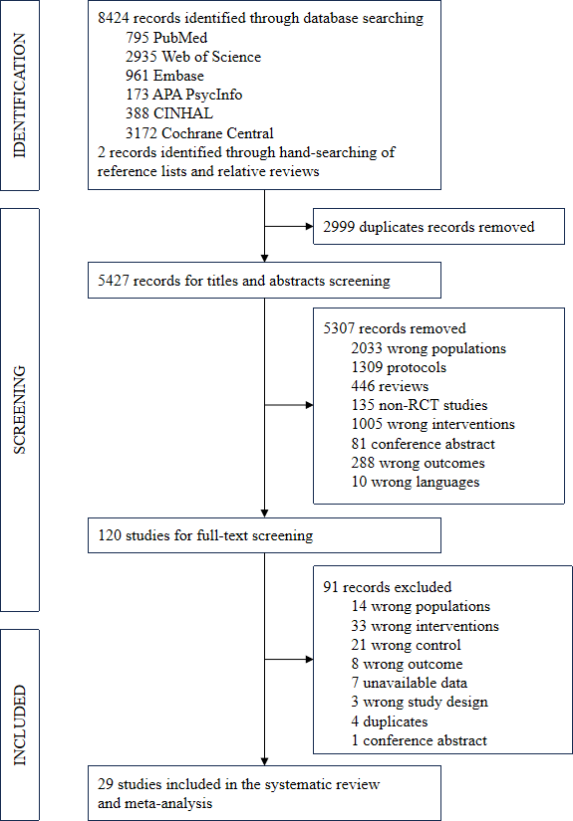
PRISMA (Preferred Reporting Items for Systematic Reviews and Meta-Analyses) flow diagram. RCT: randomized controlled trial.

The literature search across 6 databases generated 8424 articles, and an additional 2 records were added through hand-searching relevant reviews. After removing duplicates, titles and abstracts of 5427 studies were screened. Subsequently, 120 full texts were reviewed. However, 91 studies were excluded based on the eligibility criteria for the following reasons: wrong populations (n=14), wrong interventions (n=33), wrong control (n=21), wrong outcome (n=8), unavailable data (n=7), wrong study design (n=3), duplicates (n=4), and conference abstract (n=1). A detailed list of exclusions with corresponding reasons can be found in Multimedia Appendix 3. Finally, a total of 29 studies [19-23,31-54] were included in the systematic review and meta-analysis. Details are presented in the PRISMA flowchart in Figure 1.

### Study Characteristics

The details of the study characteristics are listed in Multimedia Appendix 4. The 29 studies were conducted in Spain [21,31,32], France [33], the United Kingdom [34-37], Korea [19,38,39], India [20], Australia [40-42], China [43], Japan [22], the United States of America [23,44-51], Belgium [52], New Zealand [53], and Germany [54]. Sample sizes ranged from 20 to 650 across the included studies, with the average age ranging from 22 to 55 years. Behavioral theories (ie, social cognitive theory, self-regulatory theory, control theory, operant conditioning, ecological theory, social network theory, transtheoretical model, habit formation theory, social support theory, and behavior self-management theory) were used in the design of the mobile app interventions. The intervention duration ranges from 2 months to 24 months. The intervention contents shared some common features, such as goal setting, social support, self-monitoring, and the use of credible sources. Usual care and no intervention were the primary forms of control groups. Outcome measures included weight-related outcomes (weight, BMI, WC, fat mass, and fat mass percentage), behavioral outcomes (MVPA and energy intake), and metabolic outcomes (SBP, DBP, triglycerides, and HbA_1c_).

In the 29 included RCTs, 34 BCTs from 13 categories were identified in the intervention group, while 20 BCTs from 9 categories were identified in the control group. The number of BCTs identified per study ranged from 2 to 16 (median 8, mean 8.69, SD 3.79) in the intervention group and from 0 to 14 (median 2) in the control group (see Multimedia Appendix 5). The most frequently identified BCTs in the intervention group were: 2.3 “self-monitoring of behavior” (n=25), 4.1 “instruction on how to perform the behavior” (n=24), 2.2 “feedback on behavior” (n=20), 1.1 “goal setting (behavior)” (n=19), and 1.4 “action planning” (n=15). In the control group, the most frequently used BCT was 4.1 “instruction on how to perform the behavior” (n=18). Details are shown in Table 1.

**Table 1.** Frequency of behavior change techniques used in the intervention and control group in each study.

BCT^a^ Taxonomy	Intervention group	Control group
1.1 Goal setting (behavior)	19 (65.5)	8 (27.6)
1.2 Problem solving	7 (24.1)	2 (6.9)
1.3 Goal setting (outcome)	8 (27.6)	3 (10.3)
1.4 Action planning	15 (51.7)	6 (20.7)
1.5 Review behavior goals	5 (17.2)	1 (3.4)
1.7 Review outcome goals	4 (13.8)	1 (3.4)
2.1 Monitoring of behavior by others without feedback	2 (6.9)	0 (0.0)
2.2 Feedback on behavior	20 (70.0)	2 (6.9)
2.3 Self-monitoring of behavior	25 (86.2)	7 (24.1)
2.4 Self-monitoring of outcomes of behavior	14 (48.3)	4 (13.8)
2.5 Monitoring of outcomes of behavior without feedback	1 (3.4)	0 (0.0)
2.6 Biofeedback	3 (10.3)	0 (0.0)
2.7 Feedback on outcomes of behavior	13 (44.8)	2 (6.9)
3.1 Social support (unspecified)	16 (55.2%)	4 (13.8%)
3.2 Social support (practical)	6 (20.7)	3 (10.3)
3.3 Social support (emotional)	5 (17.2)	1 (3.4)
4.1 Instruction on how to perform the behavior	24 (82.8)	18 (62.1)
5.1 Information about health consequences	7 (24.1)	5 (17.2)
6.1 Demonstration of the behavior	3 (10.3)	1 (3.4)
6.2 Social comparison	3 (10.3)	0 (0.0)
7.1 Prompts/cues	13 (44.8)	2 (6.9)
8.1 Behavioral practice/rehearsal	2 (6.9)	0 (0.0)
8.3 Habit formation	2 (6.9)	0 (0.0)
8.7 Graded tasks	2 (6.9)	0 (0.0)
10.1 Material incentive (behavior)	3 (10.3)	0 (0.0)
10.3 Nonspecific reward	4 (13.8)	0 (0.0)
10.4 Social reward	5 (17.2)	0 (0.0)
10.8 Incentive (outcome)	1 (3.4)	0 (0.0)
10.9 Self-reward	1 (3.4)	0 (0.0)
11.2 Reduce negative emotions	1 (3.4)	0 (0.0)
11.3 Conserving mental resources	4 (13.8)	1 (3.4)
12.5 Adding objects to the environment	12 (41.4)	2 (6.9)
14.4 Reward approximation	1 (3.4)	0 (0.0)
15.4 Self-talk	1 (3.4)	0 (0.0)

a BCT: behavior change technique

**Figure 2.**
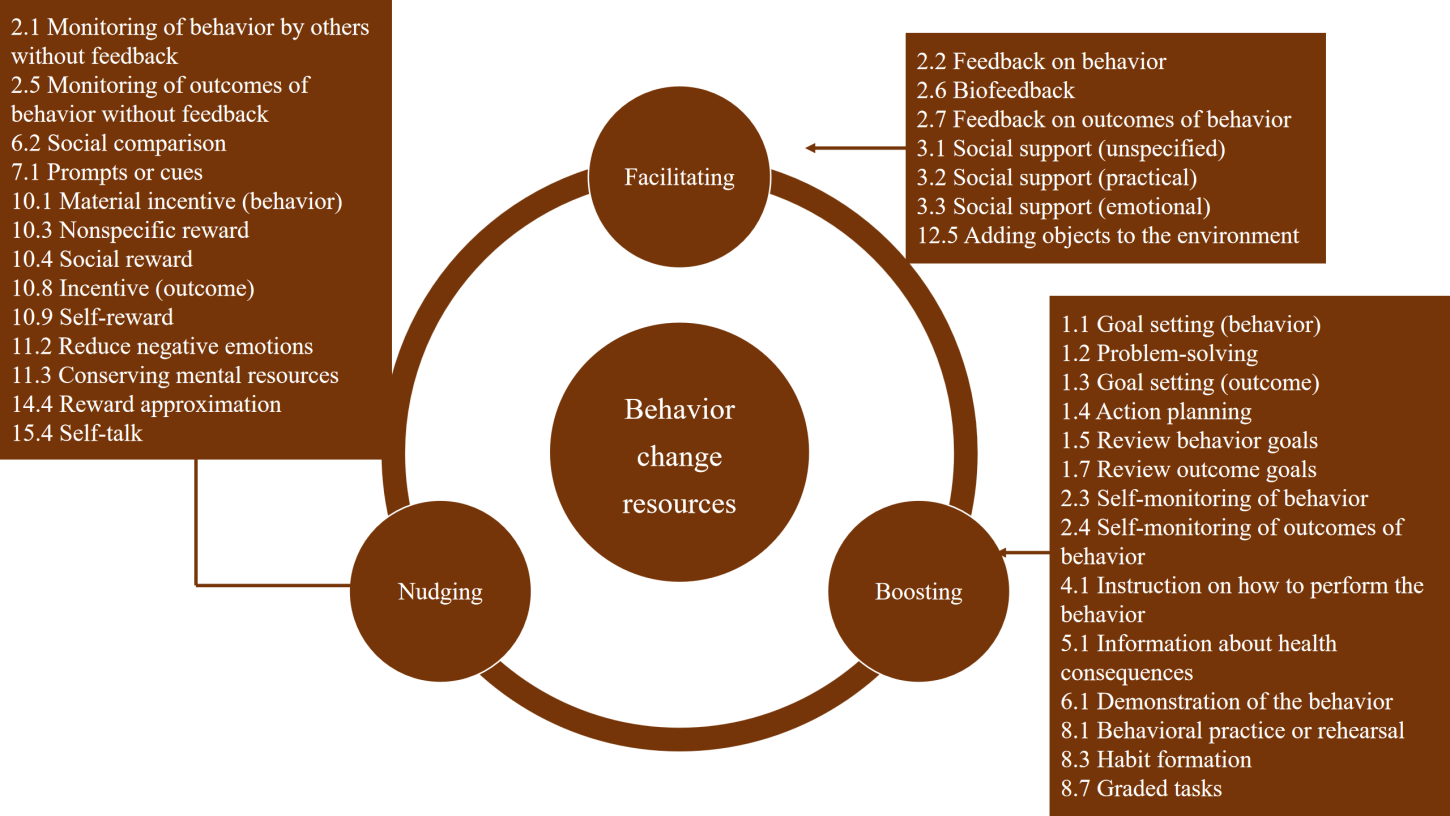
Behavior change resources were identified in each study.

The identified BCTs were categorized into 3 resource types: facilitating, boosting, and nudging (see Figure 2 and Multimedia Appendix 6). The specifics of BCRs identified in each study are presented in Table 2. Among the 29 included RCTs, 24 studies [19-22,31-34,36-38,40-44,46-52,54] used facilitating resources, 24 [19-22,31-38,40-42,44,47-54] used boosting resources, and 19 [21,22,32-38,40-42,44,45,47-50,54] used nudging resources. Fifty-nine percent of the included studies used all 3 types of resources, while only 4 studies [43,45,46,53] used 1 resource type.

**Table 2.** Behavior change resources (BCRs) identified in each study.

Studies	Facilitating	Boosting	Nudging	Total
Apiñaniz et al [31]	✓	✓		2
Bughin et al [33]	✓	✓	✓	3
Carter et al [34]	✓	✓	✓	3
Choi et al [19]	✓	✓		2
Domal et al [20]	✓	✓		2
Duncan et al [40]	✓	✓	✓	3
Godino et al [44]	✓	✓	✓	3
Hebden et al [41]	✓	✓	✓	3
Hurkmans et al [52]	✓	✓		2
Hutchesson et al [42]	✓	✓	✓	3
Jiang et al [43]	✓			1
Kliemann et al [35]		✓	✓	2
Lugones-Sanchez et al [21]	✓	✓	✓	3
Lugones-Sanchez et al [32]	✓	✓	✓	3
Nakata et al [22]	✓	✓	✓	3
Palacios et al [45]			✓	1
Patel et al [23]				0
Rogers et al [46]	✓			1
Shin et al [38]	✓	✓	✓	3
Simpson et al [36]	✓	✓	✓	3
Spring et al [47]	✓	✓	✓	3
Thomas et al [48]	✓	✓	✓	3
Vaz et al [49]	✓	✓	✓	3
Whitelock et al [37]	✓	✓	✓	3
Allen et al [50]	✓	✓	✓	3
Ross et al [51]	✓	✓		2
Jospe et al [53]		✓		1
Jin et al [39]				0
Gemesi et al [54]	✓	✓	✓	3
Total number of each BCR	24	24	19	67

### Risk of Bias and GRADE Assessment

**Figure 3.**
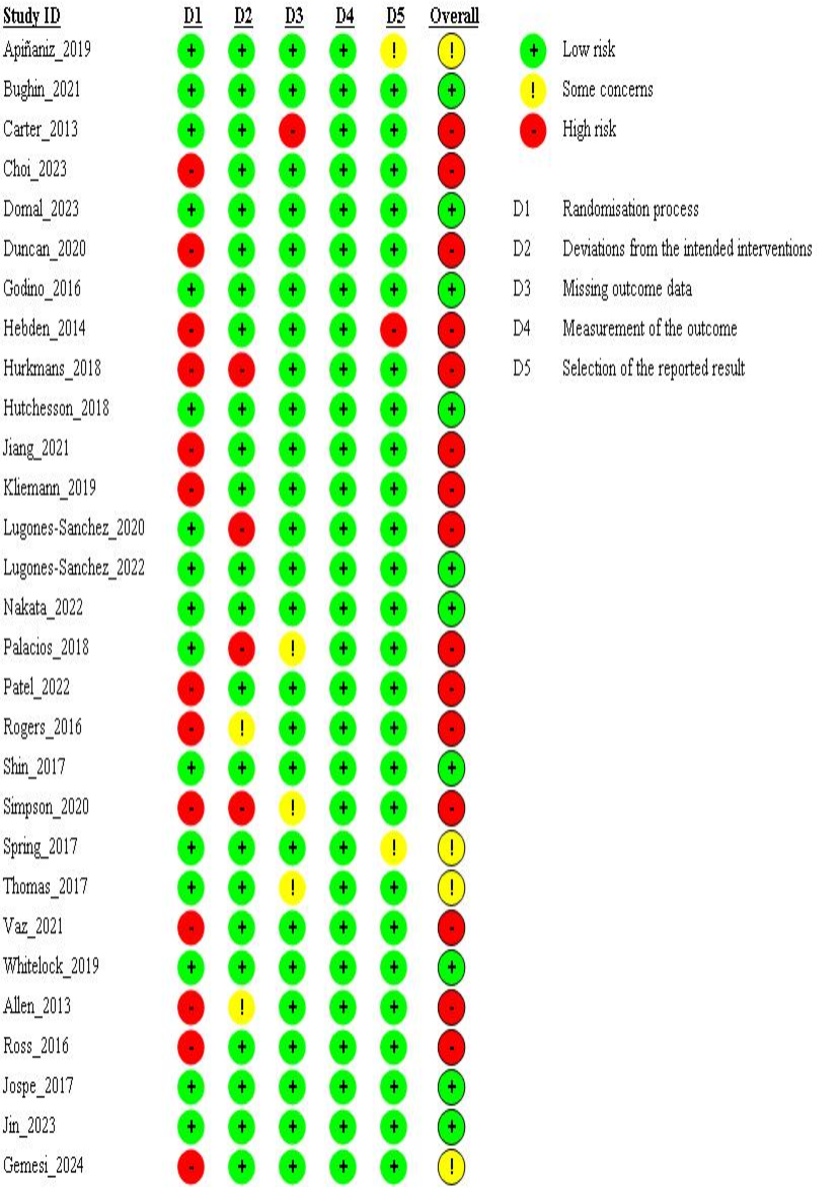
Risk of bias assessment of 29 included studies [19-23,31-54].

Of the 29 included studies, 10 [20-22,33,37-39,42,44,53] were judged to have low risk of bias, 4 [31,47,48,54] as having some concerns, and 15 [19,23,32,34-36,40,41,43,45,46,49-52] as having high risk of bias (Figure 3). While all studies reported the use of randomization, 13 studies [19,23,35,36,40,41,43,46,49-52,54] failed to explain the allocation concealment process, which was considered a high risk in this domain. Other significant sources of bias included high dropout rates with unreported reasons and the absence of appropriate analysis to assess the impact of missing outcome data.

Of the 11 outcomes included, the overall quality of evidence was assessed to be very low to moderate according to the GRADE assessment (Table 3). The risk of bias was considered serious based on the results of ROB 2 assessments. Indirectness was not considered serious, as the outcomes were directly measured. No publication bias was detected for any of the outcomes. Inconsistency ranged from not serious to very serious based on the heterogeneity values, and imprecision ranged from not serious to serious, as determined by the 95% CI.

**Table 3.** Grading of Recommendation, Assessment, Development, and Evaluation assessment.

Outcome	Studies, n/N (%)	Quality assessment					Patients (I^a^/C^b^), n/N	Effect, mean difference (95% CI)	Quality
		Risk of bias	Inconsistency	Indirectness	Imprecision	Publication bias			
Weight	28/29 (97)	Serious	Very Serious	Not serious	Not serious	Undetected	1743/1680	−1.45 (−2.01 to −0.89)	Very Low
BMI	18/29 (62)	Serious	Serious	Not serious	Not serious	Undetected	1241/1216	−0.35 (−0.57 to −0.13)	Low
WC^c^	12/29 (41)	Serious	Very serious	Not serious	Serious	Undetected	897/877	−1.98 (−3.42 to −0.55)	Very Low
Fat mass	10/29 (34)	Serious	Serious	Not serious	Not serious	Undetected	537/521	−1.32 (−1.94 to −0.69)	Low
Fat mass percentage	11/29 (38)	Serious	Very serious	Not serious	Not serious	Undetected	630/632	−0.40 (−1.00 to 0.19)	Very Low
MVPA^d^	6/29 (21)	Serious	Not serious	Not serious	Serious	Undetected	319/248	−0.69 (−5.67 to 4.28)	Low
Energy intake	6/29 (21)	Serious	Very serious	Not serious	Serious	Undetected	619/573	−62.72 (−181.62 to 56.18)	Very Low
SBP^e^	9/29 (31)	Serious	Very serious	Not serious	Serious	Undetected	493/502	−0.14 (−2.66 to 2.37)	Very Low
DBP^f^	8/29 (28)	Serious	Very serious	Not serious	Not serious	Undetected	460/469	−1.76 (−3.47 to −0.04)	Very Low
Triglycerides	6/29 (21)	Serious	Not serious	Not serious	Not serious	Undetected	294/293	0.06 (−0.19 to 0.31)	Moderate
HbA_1c_^g^	5/29 (17)	Serious	Very serious	Not serious	Not serious	Undetected	316/275	−0.13 (−0.22 to −0.04)	Very Low

a I: intervention group.

b C: control group.

c WC: waist circumference.

d MVPA: moderate-to-vigorous physical activity.

e SBP: systolic blood pressure.

f DBP: diastolic blood pressure.

g HbA_1c_: hemoglobin A_1c_.

### Effects of Mobile Phone App

The review examined the effects of mobile phone app interventions on weight-related outcomes (weight, BMI, WC, fat mass, and fat mass percentage), behavioral outcomes (MVPA and energy intake), and metabolic outcomes (SBP, DBP, triglycerides, and HbA_1c_). A summary of the meta-analyses’ results was shown in Table 4.

**Table 4.** A summary of meta-analyses results on each outcome.

Outcomes	Studies, n/N (%)	Sample size (intervention group), n	Sample size (control group), n	Mean difference (95% CI)	*P* value	*I^2^* (%)
Weight-related outcomes						
Weight (kg)	28/29 (97)	1743	1680	−1.45 (−2.01 to −0.89)	<.001	56
BMI (kg/m^2^)	18/29 (62)	1241	1216	−0.35 (−0.57 to −0.13)	.002	43
WC^a^ (cm)	12/29 (41)	897	877	−1.98 (−3.42 to −0.55)	.007	82
Fat mass (kg)	10/29 (34)	537	521	−1.32 (−1.94 to −0.69)	<.001	31
Fat mass percentage (%)	11/29 (38)	630	632	−0.40 (−1.00 to 0.19)	.18	60
Behavioral outcomes						
MVPA^b^ (mins/day)	6/29 (21)	319	248	−0.69 (−5.67 to 4.28)	.78	0
Energy intake (kcal/day)	6/29 (21)	619	573	−62.72 (−181.62 to 56.18)	.30	62
Metabolic outcomes						
SBP^c^ (mm Hg)	9/29 (31)	493	502	−0.14 (−2.66 to 2.37)	.91	66
DBP^d^ (mm Hg)	8/29 (28)	460	469	−1.76 (−3.47 to −0.04)	.04	61
Triglycerides (mmol/L)	6/29 (21)	294	293	0.06 (−0.19 to 0.31)	.64	9
HbA_1c_^e^ (%)	5/29 (17)	316	275	−0.13 (−0.22 to -0.04)	.005	82

a WC: waist circumference.

b MVPA: moderate-to-vigorous physical activity.

c SBP: systolic blood pressure.

d DBP: diastolic blood pressure.

e HbA_1c_: hemoglobin A_1c_.

#### Weight-Related Outcomes

##### Weight

**Figure 4.**
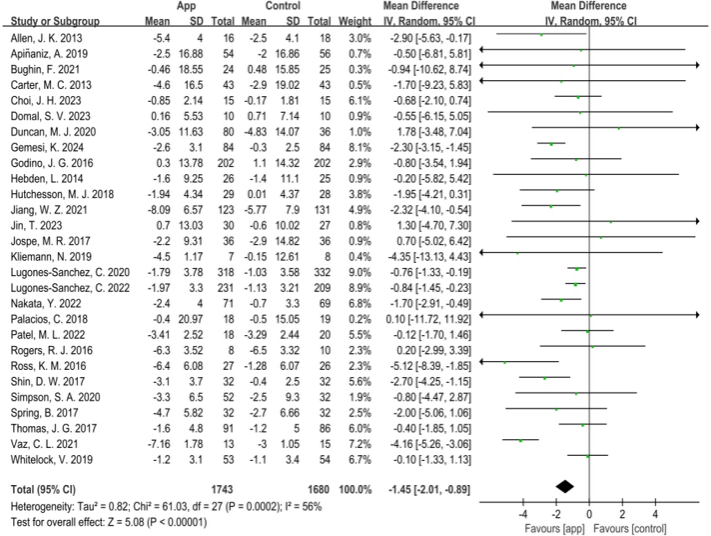
Meta-analysis of weight [19-23,31-51,53,54].

Twenty-nine RCTs [19-23,31-54] measured weight as an outcome, but 1 study [52] was excluded from the meta-analysis due to unavailable outcome data. Therefore, the outcomes of 28 studies involving 1743 participants in the intervention group and 1680 participants in the control group were pooled. The results indicated that mobile phone app interventions significantly reduced weight (MD=−1.45 kg, 95% CI −2.01 to −0.89; *P*<.001), with modest heterogeneity (*Χ*^*2*^=61.03, df=27, *I^2^*=56%), as determined by the random-effects model (Figure 4).

##### BMI

Eighteen studies [19-21,32-34,36,38,39,41-46,50,52,53] assessing BMI were included in the meta-analysis. The mobile phone app intervention resulted in a significant reduction in BMI (MD=−0.35 kg/m^2^, 95% CI −0.57 to −0.13; *P*=.002), with moderate heterogeneity (*Χ*^*2*^=29.91, df=17, *I^2^*=43%; Multimedia Appendix 7).

##### WC

Twelve studies [20,21,38-40,42-44,46,49,50,53] with 897 participants in the intervention group were included in the meta-analysis to examine the effects of mobile phone app intervention on WC. The results showed that WC was significantly reduced (MD=−1.98 cm, 95% CI −3.42 to −0.55; *P*=.007), with high heterogeneity (*Χ*^*2*^=62.49, df=11, *I^2^*=82%; Multimedia Appendix 8).

##### Fat Mass

The effect on fat mass was measured by 11 RCTs [19,20,32,33,38,39,42,43,46,53,54], with a total of 1058 participants pooled for the meta-analysis. Fat mass was significantly reduced (MD=−1.32 kg, 95% CI −1.94 to −0.69; *P*<.001) due to the mobile app intervention. Heterogeneity was low across the included studies (*Χ*^*2*^=13.11, df=9, *I^2^*=31%; Multimedia Appendix 9).

##### Fat Mass Percentage

Eleven RCTs [19,20,32-34,37,38,42,43,46,54] were included to examine the effects on fat mass percentage. The mobile app intervention did not lead to a statistically significant reduction in fat mass percentage (MD=−0.40, *P*=.18, 95% CI −1.00 to 0.19), and the heterogeneity was moderate (*Χ*^*2*^=25.08, df=10, *I^2^*=60%; Multimedia Appendix 10).

### Behavioral Outcomes (MVPA and Energy Intake)

The mobile phone app intervention decreased MVPA, but this change was not statistically significant (MD=−0.69, 95% CI −5.67 to 4.28; *P*=.78; Multimedia Appendix 11). No heterogeneity was observed. When pooling the data on energy intake in the meta-analysis, the results indicated a nonsignificant effect size (MD=−62.72 kcal/day, 95% CI −181.62 to 56.18; *P*=.30) with moderate heterogeneity (*Χ*^*2*^=13.33, df=5, *I^2^*=62%; Multimedia Appendix 12).

### Metabolic Outcomes (SBP, DBP, Triglycerides, and HbA_1c_)

SBP was nonsignificantly reduced (MD=−0.14 mm Hg, 95% CI −2.66 to 2.37; *P*=.91). The mobile phone app intervention significantly decreased DBP (MD=−1.76 mm Hg, 95% CI −3.47 to −0.04; *P*=.04). It led to a nonsignificant increase in triglyceride levels (MD=0.06 mmol/L, 95% CI −0.19 to 0.31; *P*=.64). In addition, there was a significant decrease in HbA_1c_ levels (MD=−0.13%, 95% CI −0.22 to −0.04; *P*=.005), with high heterogeneity (*Χ*^*2*^=22.79, df=4, *I^2^*=82%; Figures S7-S10 in Multimedia Appendices 13-16).

### Subgroup and Sensitivity Analysis of Weight

Five subgroup analyses were conducted to explore potential factors influencing the effects of the apps, intervention content (single intervention [diet or PA] versus combined intervention [diet+PA]), theory presence (theory-based versus nontheory-based), intervention duration (short-term [≤3 months] versus medium-term [6 months] versus long-term [>6 months]), number of BCRs used (3 resources vs <3 resources), and number of BCTs used (<median [8] vs ≥median [8]).

Among these, the combined intervention demonstrated a greater effect size (MD=−1.82, 95% CI −2.48 to −1.16 kg) compared with the single intervention group (MD=−0.24, 95% CI −1.00 to 0.53 kg), with statistical significance. Regarding intervention duration, the medium-term (6 mo) group exhibited the largest effects (MD=−2.50, 95% CI −3.65 to −1.35 kg), significantly outperforming the other two groups. In addition, the number of BCTs used ≥8 had a greater effect size (MD=−1.83, 95% CI −2.56 to −1.09 kg vs MD=−0.61, 95% CI −1.28 to −0.07 kg; *P=*.02; Table 5).

**Table 5.** Subgroup analyses.

Variables and subgroups		Number of studies	MD^a^ (95% CI)	*I^2^* (%)	*P* value of subgroup difference
Intervention contents					.002
Single intervention (diet or PA)		9	−0.24 (−1.00 to 0.53)	0	
Combined intervention (diet+PA^b^)		19	−1.82 (−2.48 to –1.16)	64	
Theory					.34
Theory-based		8	−0.97 (−1.96 to 0.02)	0	
Nontheory-based		20	−1.54 (−2.20 to –0.89)	65	
Intervention duration					.04
Short-term (≤3 months）		14	−1.12 (−1.61 to −0.62)	36	
Medium-term (6 months)		10	−2.50 (−3.65 to −1.35)	42	
Long-term (＞6 months)		4	−0.47 (−1.65 to 0.71)	0	
BCRs^c^					.44
=3 resources		17	−1.56 (−2.23 to –0.88)	67	
<3 resources		11	−1.10 (−2.06 to –0.13)	14	
BCTs^d^					.02
<8		12	−0.61 (−1.28 to 0.07)	0	
≥8		16	−1.83 (−2.56 to –1.09)	70	

a MD: mean difference.

b PA: physical activity.

c BCR: behavior change resources.

d BCT: behavior change techniques.

Sensitivity analyses were performed using the leave-one-out and high-risk study exclusion methods. Notably, after excluding one study from the meta-analysis, the weight reduction effect size changed from −1.45 kg (95% CI −2.01 to −0.89) to −1.20 kg (95% CI −1.61 to −0.79), indicating that this study had a relatively significant influence on the overall effect size [49]. The heterogeneity also decreased, from 56% to 23%. In addition, excluding high-risk studies resulted in a weight reduction of −1.24 kg (95% CI −1.84 to −0.83) and an *I^2^* of 36%. High-risk studies may overestimate the effect size. However, the sensitivity analyses demonstrate that the results are robust, as no statistically significant changes were observed. This specific study and high-risk studies may account for the heterogeneity to some extent.

### Publication Bias

**Figure 5.**
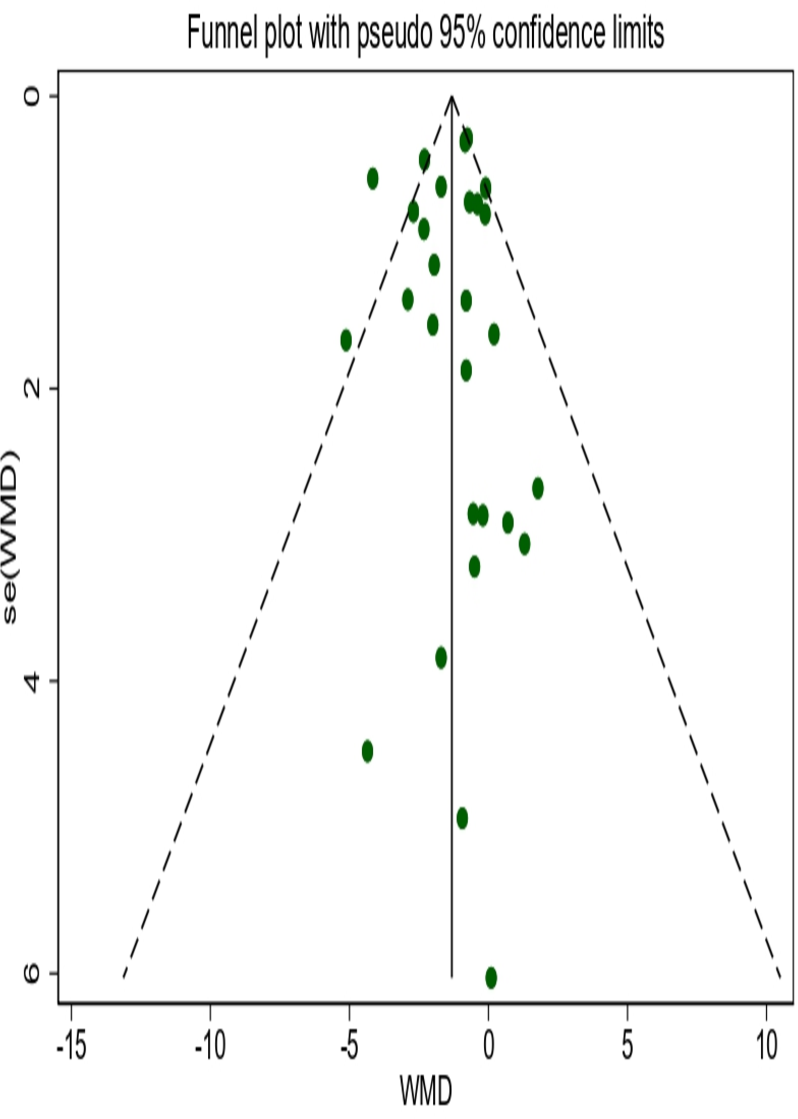
Funnel plot of weight. WMD: weighted mean difference.

Funnel plot and Egger test for publication bias were conducted when a minimum of 10 studies were incorporated into the meta-analysis. No significant publication bias was detected for weight (Egger test, *t*_27_=−0.28; *P*=.78), BMI (Egger test, *t*_17_=0.63; *P*=.54), WC (Egger test, *t*_11_=0.10; *P*=.92), fat mass (Egger test, *t*_9_=0.54; *P*=.61), or fat mass percentage (Egger test, *t*_10_=−0.69;* P*=.51). The funnel plots were displayed in Figure 5 and Figure S11-S14 in Multimedia Appendices 17-20.

## Discussion

### Principal Findings

A total of 29 RCTs [19-23,31-54] were included in this systematic review and meta-analysis, examining 11 outcomes. The results of this review indicated that mobile app interventions significantly reduced weight, BMI, WC, fat mass, DBP, and HbA_1c_. However, nonsignificant effects were observed for other outcomes, including fat mass percentage, behavioral outcomes (ie, MVPA and energy intake), and metabolic outcomes (ie, SBP and triglycerides). BCTs and BCRs used in the included studies were also identified, with most studies (59%) using 3 resource types (ie, facilitating, boosting, and nudging). In addition, intervention content, duration, and the number of BCTs used were identified as influencing factors through subgroup analyses.

### Interpretation of Findings

#### Effects of Mobile Phone App on Weight-Related Outcomes

The meta-analysis suggested that interventions delivered via mobile phone apps significantly reduced weight among adults with overweight and obesity, which is consistent with findings from previous meta-analyses [14,16]. Notably, in our meta-analysis, the effect size for weight loss was 1.45 kg, while meta-analyses conducted by Chew et al [16] and Antoun et al [14] reported weight loss values of 2.55 kg and 1.99 kg, respectively. The main reason for this difference may be that the two aforementioned reviews included interventions involving both mobile phone apps and nonmobile interventions, whereas our meta-analysis focused on studies using interventions delivered solely by mobile apps or applications combined with nonmobile interventions [55]. This suggests that, compared with app interventions alone, the combination of app interventions with other strategies, such as health coaching, is more effective. Overall, mobile app–based interventions are a promising and cost-effective way of supporting weight loss.

According to the results of the subgroup analyses, the combined intervention group (diet+PA) showed better outcomes than the single intervention group (diet or PA), which is consistent with the results of previous studies [56,57]. These findings suggest that future researchers should design weight-loss apps targeting both diet and PA, rather than focusing on a single behavior change. Intervention duration is also a significant factor influencing the effects, with the greatest reductions observed in the medium term. This can be explained by the phenomenon where the effects of mobile apps show initial improvements, peak at 6 months, and then gradually decrease due to a decline in behavior change adherence over time. Therefore, the sustainability of weight-loss effects through the effective use of behavior change strategies is crucial for successful weight loss.

High BMI is associated with various conditions, including cardiovascular disease (CVD), certain cancers, and breathing problems [58]. BMI was also significantly reduced in our meta-analysis. This finding contrasts with the results of a previous meta-analysis [16], which may be attributed to the discrepancy in the number of included original studies (18 vs 3). Therefore, our meta-analysis may provide a more comprehensive insight.

WC, a reliable indicator of not only total body fat but also abdominal visceral fat [59], is an accurate predictor of disease [60]. It exhibits superior efficacy as a marker for all-cause mortality, the incidence of cardiovascular disease [61], and metabolic syndrome [62] compared with BMI [63-65], which solely represents total body fat mass. This association between WC and these health outcomes can be explained by the cellular mechanisms linking metabolic syndrome to a proinflammatory state, with visceral obesity playing a central role. In addition, visceral obesity disrupts the normal physiological balance of adipokines, insulin resistance, and endothelial dysfunction, creating a proatherogenic state. Therefore, visceral obesity is closely associated with the incidence of cardiovascular disease [66]. Given the importance of WC in clinical practice, we consider it a critical outcome. However, among the 29 included studies, only 12 (41%) RCTs reported the effects of mobile apps on WC. We recommend incorporating WC as an outcome in future original studies.

After combining data from 12 RCTs in the meta-analysis, WC was significantly reduced through the use of weight loss apps among individuals with overweight and obesity, consistent with a review performed by Cai et al [15], but inconsistent with a previous meta-analysis by Chew et al [67]. This discrepancy may be explained by the fact that only four RCTs were included in Chew et al’s review [67] to examine the effects on WC, 2 of which focused on the older population. This may have reduced the effects of mobile phone apps on WC, as basal metabolic rate decreases almost linearly with age [68], making it more difficult for older adults to lose weight.

Our meta-analysis also identified fat mass and fat mass percentage as primary outcomes, revealing a significant reduction in fat mass but not in fat mass percentage. This finding suggests that body components other than fat, such as lean muscle mass and bone mass, may have also decreased during the weight loss period. However, these components are crucial for body functions and overall health. Therefore, future research should focus on strategies aimed specifically at fat reduction, such as individualized fat-loss plans and physical fitness training.

#### Effects of Mobile Phone App on Behavioral and Metabolic Outcomes

Lifestyle modification, including increasing physical activity and restricting calories, is considered the cornerstone of obesity management [69,70]. Based on the effectiveness of weight loss apps observed in our meta-analysis, it is worth discussing the underlying reasons behind these lifestyle interventions. Surprisingly, an increase in MVPA was not observed, which may be attributed to several factors. Many weight loss apps are designed to help users self-monitor their PA rather than actively encourage PA [15,21,32]. Our review found that using mobile phone apps can reduce energy intake by approximately 100 kcal/day, which may partly explain the effectiveness of weight loss. However, this reduction was not statistically significant. We recommend that behavior change strategies be more thoroughly considered in the design of future mobile app–based interventions.

In our meta-analysis, we explored the effects on four outcomes (ie, SBP, DBP, triglycerides, and HbA_1c_) and observed significant reductions in DBP and HbA_1c_. However, these reductions did not result in clinically significant benefits. This phenomenon may be explained by two factors. First, reductions in blood pressure, triglycerides, and HbA_1c_ are influenced by various factors (eg, pharmacological treatment, diet, and PA) and are not solely dependent on weight management. Weight loss mobile apps are not specifically designed to lower blood pressure, triglycerides, or HbA_1c_. As a result, the intervention strategies within the weight loss app may not be sufficient to meet the requirements for reducing blood pressure, lowering triglycerides, and improving HbA_1c_ levels. Given that individuals with overweight or obesity are at higher risk of developing metabolic syndromes [62], the app could be redesigned to better serve the needs of this high-risk population.

Second, while weight reduction was statistically significant in our review, the extent of weight loss may be insufficient to achieve a clinically meaningful reduction in metabolic outcomes [71,72]. A previous study [73] demonstrated that a 5% weight loss significantly decreases plasma concentrations (eg, glucose, insulin, triglycerides, and leptin) of certain risk factors for cardiometabolic disease. A systematic review [74] also indicated that for every 10 kg reduction in weight, there is a corresponding decrease of 6.0 mm Hg in SBP and 4.6 mm Hg in DBP levels. However, due to the limited number of RCTs investigating the effects on metabolic outcomes, our meta-analysis is based on a relatively small number of original studies. Given the importance of clinical significance in weight loss, more RCTs that include assessments of metabolic outcomes are needed to clarify the relationship between weight loss and metabolic outcomes.

#### Interpretation of Findings of BCTs and BCRs

To our knowledge, this is the first review to identify BCRs used in mobile app interventions for adults with overweight and obesity. The classification standards were based on the BCRM proposed by Michaelsen in 2022 [26]. Our findings indicate that 59% of studies used 3 resource types in their intervention designs, 21% used 2 types, 14% used 1 type, and 2 studies did not use any resources. Standardization of these classification standards is needed for the future, and further exploration of optimal resource combinations is warranted. Based on the results of the subgroup analyses, the group using ≥8 BCTs showed better outcomes than the group using <8 BCTs, suggesting that a higher number of BCTs was associated with better effect sizes to some extent. Future studies should explore more effective combinations of BCTs to enhance their effects.

### Strengths and Limitations

Several strengths can be identified in this review. First, a thorough examination of the effects of mobile phone apps on weight loss among adults with overweight and obesity was conducted. Our meta-analysis includes 11 outcomes based on findings from 29 studies. Weight-related outcomes reflect the direct effects of mobile apps, metabolic outcomes provide evidence of the association between weight reduction and health benefits, and behavioral outcomes inform researchers about the extent to which current interventions target diet and PA, offering guidance for future intervention design. This comprehensive set of outcomes offers substantial insights into the current effectiveness and status of mobile apps in weight loss interventions. Second, only RCTs were included in this review, as they are considered the gold standard in clinical research. This selection criterion ensures that our findings are based on high-quality studies, minimizing bias and enhancing the reliability of the results. Third, the BCTs used in the included RCTs were identified. Systematically reviewing the use of BCTs contributes to the standardization of intervention strategies and enhances comparability across studies, especially given the considerable methodological variability in current weight loss interventions. This provides a reference for researchers to replicate, refine, or design new intervention approaches. Fourth, mapping BCTs to BCRs based on the BCRM helps clarify the resources required to implement specific BCTs during intervention design. This approach also supports more tailored and practical intervention strategies by taking into account the resources available to patients, thereby promoting interventions that are both targeted and feasible.

However, there are also some limitations in this study. First, we only included adults with overweight and obesity without pre-existing medical conditions, which may limit the generalizability of the results to broader populations, such as individuals with diabetes or CVD. However, adults with overweight and obesity without medical conditions represent a critical population from the perspective of preventive medicine, as they are at high risk of developing chronic diseases. Early interventions targeting this population group could potentially reduce the burden of future disease onset. Moreover, there are notable differences in intervention responsiveness and health needs between adults with overweight and obesity with and without medical conditions. By including only participants without comorbidities, this study allows for a more focused analysis of lifestyle-based mobile app interventions, without the influence of complex confounding factors such as medication use or the bidirectional interactions between obesity and chronic diseases. This approach allows for a clearer assessment of behavior change outcomes, especially in the identification of BCTs and the associated BCRs. Second, we identified the BCTs and BCRs used in the RCT interventions included in this review. Although interrater agreement in coding the BCTs and resources was high, the coding process inherently involves a degree of subjectivity, which may influence the results related to BCTs and BCRs. Furthermore, given the relatively limited app of the BCRM in previous research, standardized procedures for mapping BCTs to corresponding resources have not yet been established. The mapping criteria proposed in this study, therefore, require further testing and validation in future research. Finally, the effectiveness of weight loss was influenced not only by the quantity of BCTs and resources used but also by their quality, including factors such as the intensity, frequency, and tailoring of interventions to individual needs. As a result, no meta-regression was conducted to link specific BCTs or BCRs to their effectiveness. We advocate for the conducting of more RCTs to better clarify the specific effects of individual BCT or BCR.

### Conclusions

This meta-analysis presents findings that demonstrate that mobile phone app interventions significantly reduce weight, BMI, WC, fat mass, DBP, and HbA_1c_ among adults with overweight and obesity. These mobile phone app interventions are cost-effective and can be applied to a large population. However, current mobile apps have not achieved clinically significant weight loss. Future studies should focus on optimizing app interventions by incorporating more effective behavior change strategies and resources to enhance their overall effectiveness.

## Supplementary material

10.2196/63313 Multimedia Appendix 1 Search strategy.

10.2196/63313 Multimedia Appendix 2 Taxonomy of behavioral change techniques.

10.2196/63313 Multimedia Appendix 3 Reasons for exclusion of studies.

10.2196/63313 Multimedia Appendix 4 Characteristics of the included studies.

10.2196/63313 Multimedia Appendix 5 Behavioral change techniques identified in each study.

10.2196/63313 Multimedia Appendix 6 Mapping behavioral change techniques to behavioral change resources.

10.2196/63313 Multimedia Appendix 7 Meta-analysis of BMI.

10.2196/63313 Multimedia Appendix 8 Meta-analysis of waist circumference.

10.2196/63313 Multimedia Appendix 9 Meta-analysis of fat mass.

10.2196/63313 Multimedia Appendix 10 Meta-analysis of fat mass percentage.

10.2196/63313 Multimedia Appendix 11 Meta-analysis of moderate-to-vigorous physical activity.

10.2196/63313 Multimedia Appendix 12 Meta-analysis of energy intake.

10.2196/63313 Multimedia Appendix 13 Meta-analysis of systolic blood pressure.

10.2196/63313 Multimedia Appendix 14 Meta-analysis of diastolic blood pressure.

10.2196/63313 Multimedia Appendix 15 Meta-analysis of triglycerides.

10.2196/63313 Multimedia Appendix 16 Meta-analysis of hemoglobin A_1c_.

10.2196/63313 Multimedia Appendix 17 Funnel plot of BMI.

10.2196/63313 Multimedia Appendix 18 Funnel plot of fat mass percentage.

10.2196/63313 Multimedia Appendix 19 Funnel plot of waist circumference.

10.2196/63313 Multimedia Appendix 20 Funnel plot of fat mass.

10.2196/63313 Checklist 1 PRISMA (Preferred Reporting Items for Systematic Reviews and Meta-Analyses) checklist.

## Abbreviations

BCR: behavior change resource
BCRM: behavior change resource model
BCT: Behavior change techniques
CVD: cardiovascular disease
DBP: diastolic blood pressure
GDP: gross domestic product
GRADE framework: Grading of Recommendations, Assessment, Development, and Evaluation
HbA_1c_: hemoglobin A_1c_
MD: mean difference
MeSH: Medical Subject Headings
mHealth: mobile health
MVPA: moderate-to-vigorous physical activity
PA: physical activity
PICOS: Participants, Interventions, Comparisons, Outcomes, and Study Design framework
PRISMA: Preferred Reporting Items for Systematic Reviews and Meta-Analyses
PROSPERO: Prospective Register of Systematic Reviews
RCT: randomized controlled trial
ROB 2: Cochrane risk-of-bias version 2
SBP: systolic blood pressure
WC: waist circumference
