# Supplementary material for: Behavior Change Resources Used in Mobile App–Based Interventions Addressing Weight, Behavioral, and Metabolic Outcomes in Adults With Overweight and Obesity: Systematic Review and Meta-Analysis of Randomized Controlled Trials
Source: JMIR Mhealth Uhealth. 2025 Aug 19;13:e63313. doi: 10.2196/63313 (PMC12392691; doi:10.2196/63313)
Supplement: Multimedia Appendix 1 [file mhealth-v13-e63313-s001.docx]

Table S1 Search strategy

From 2010/1/1 to 2024/5/20

| PubMed | |
| --- | --- |
| #1 | "obesity"[MeSH Terms] OR "overweight"[MeSH Terms] OR "body weight"[Title/Abstract] OR "skinfold thickness"[Title/Abstract] OR "Bariatrics"[Title/Abstract] OR "Obese"[Title/Abstract] OR "Adiposity"[Title/Abstract] OR "abdominal fat"[Title/Abstract] OR "body fat*"[Title/Abstract] |
| #2 | "mobile applications"[MeSH Terms] OR "Smartphone"[MeSH Terms] OR "telemedicine"[MeSH Terms] OR "wearable electronic devices"[MeSH Terms] OR "mHealth"[Title/Abstract] OR "m-Health"[Title/Abstract] OR "mobile health"[Title/Abstract] OR "mobile apps"[Title/Abstract] OR "Apps"[Title/Abstract] OR "smartphone app*"[Title/Abstract] OR "telehealth"[Title/Abstract] OR "eHealth"[Title/Abstract] OR "e-Health"[Title/Abstract] OR "health monitor"[Title/Abstract] OR "tablet computers"[Title/Abstract] OR "mobile technolog*"[Title/Abstract] OR "activity tracker"[Title/Abstract] OR "activity monitor"[Title/Abstract] OR "fitbit*"[Title/Abstract] |
| #3 | "randomized controlled trial"[Publication Type] OR "randomized"[Title/Abstract] OR "placebo"[Title/Abstract] |
| #4 | #1 AND #2 AND #3 |

| Embase | |
| --- | --- |
| #1 | 'obesity'/exp OR obesity |
| #2 | 'overweight'/exp OR overweight |
| #3 | 'body weight':ab,ti OR 'skinfold thickness':ab,ti OR 'bariatrics':ab,ti OR 'obese':ab,ti OR 'adiposity':ab,ti OR 'abdominal fat':ab,ti OR 'body fat*':ab,ti |
| #4 | #1 OR #2 OR #3 |
| #5 | ‘mobile applications’:ab,ti OR ‘smartphone’:ab,ti OR ‘telemedicine’:ab,ti OR ‘Wearable Electronic Devices’:ab,ti OR ‘mHealth’:ab,ti OR ‘m-Health’:ab,ti OR ‘mobile health’:ab,ti OR ‘mobile apps’:ab,ti OR ‘Apps’:ab,ti OR ‘Smartphone app*’:ab,ti OR ‘telehealth’:ab,ti OR ‘eHealth’:ab,ti OR ‘e-Health’:ab,ti OR ‘Health monitor’:ab,ti OR ‘tablet computers’:ab,ti OR ‘mobile technolog*’:ab,ti OR ‘activity tracker’:ab,ti OR ‘activity monitor’:ab,ti OR ‘fitbit*’:ab,ti |
| #6 | 'randomized controlled trial':ab,ti OR 'randomized':ab,ti OR 'rct':ab,ti OR 'placebo':ab,ti |
| #7 | #4 AND #5 AND #6 |

| CENTRAL | |
| --- | --- |
| #1 | (Obesity):ab,ti,kw OR (overweight):ab,ti,kw OR (Body Weight):ab,ti,kw OR (Skinfold Thickness):ab,ti,kw OR (Bariatrics):ab,ti,kw OR (Obese):ab,ti,kw OR (Adiposity):ab,ti,kw OR (Abdominal fat):ab,ti,kw OR (Body fat*):ab,ti,kw |
| #2 | (mobile applications):ab,ti,kw OR (smartphone):ab,ti,kw OR (telemedicine):ab,ti,kw OR (Wearable Electronic Devices):ab,ti,kw OR (mHealth):ab,ti,kw OR (m-Health):ab,ti,kw OR (mobile health):ab,ti,kw OR (mobile apps):ab,ti,kw OR (Apps):ab,ti,kw OR (Smartphone app*):ab,ti,kw OR (telehealth):ab,ti,kw OR (eHealth):ab,ti,kw OR (e-Health):ab,ti,kw OR (Health monitor):ab,ti,kw OR (tablet computers):ab,ti,kw OR (mobile technolog*):ab,ti,kw OR (activity tracker):ab,ti,kw OR (activity monitor):ab,ti,kw OR (fitbit*):ab,ti,kw |
| #3 | (randomized controlled trial):ab,ti,kw OR (randomized):ab,ti,kw OR (RCT):ab,ti,kw OR (placebo):ab,ti,kw |
| #5 | #1 AND #2 AND #3 |

| Web of Science | |
| --- | --- |
| #1 | TS= (Obesity OR overweight OR Body Weight OR Skinfold Thickness OR Bariatrics OR Obese OR Adiposity OR Abdominal fat OR Body fat*) |
| #2 | TS= (mobile applications OR smartphone OR telemedicine OR Wearable Electronic Devices OR mHealth OR m-Health OR mobile health OR mobile apps OR Apps OR Smartphone app* OR telehealth OR eHealth OR e-Health OR Health monitor OR tablet computers OR mobile technolog* OR activity tracker OR activity monitor OR fitbit*) |
| #3 | TS= (randomized controlled trial OR randomized OR placebo OR RCT) |
| #4 | #1 AND #2 AND #3 |

| PsycINFO | |
| --- | --- |
| #1 | TI (Obesity or overweight or Body Weight or Skinfold Thickness or Bariatrics or Obese or Adiposity or Abdominal fat or Body fat*) OR AB (Obesity or overweight or Body Weight or Skinfold Thickness or Bariatrics or Obese or Adiposity or Abdominal fat or Body fat*) |
| #2 | TI (mobile applications OR smartphone OR telemedicine OR Wearable Electronic Devices OR mHealth OR m-Health OR mobile health OR mobile apps OR Apps OR Smartphone app* OR telehealth OR eHealth OR e-Health OR Health monitor OR tablet computers OR mobile technolog* OR activity tracker OR activity monitor OR fitbit*) OR AB (mobile applications OR smartphone OR telemedicine OR Wearable Electronic Devices OR mHealth OR m-Health OR mobile health OR mobile apps OR Apps OR Smartphone app* OR telehealth OR eHealth OR e-Health OR Health monitor OR tablet computers OR mobile technolog* OR activity tracker OR activity monitor OR fitbit*) |
| #3 | TI (randomized controlled trial or randomized or placebo or RCT) OR AB (randomized controlled trial or randomized or placebo or RCT) |
| #4 | #1 AND #2 AND #3 |

| CINAHL | |
| --- | --- |
| #1 | TI (Obesity or overweight or Body Weight or Skinfold Thickness or Bariatrics or Obese or Adiposity or Abdominal fat or Body fat*) OR AB (Obesity or overweight or Body Weight or Skinfold Thickness or Bariatrics or Obese or Adiposity or Abdominal fat or Body fat*) |
| #2 | TI (mobile applications OR smartphone OR telemedicine OR Wearable Electronic Devices OR mHealth OR m-Health OR mobile health OR mobile apps OR Apps OR Smartphone app* OR telehealth OR eHealth OR e-Health OR Health monitor OR tablet computers OR mobile technolog* OR activity tracker OR activity monitor OR fitbit*) OR AB (mobile applications OR smartphone OR telemedicine OR Wearable Electronic Devices OR mHealth OR m-Health OR mobile health OR mobile apps OR Apps OR Smartphone app* OR telehealth OR eHealth OR e-Health OR Health monitor OR tablet computers OR mobile technolog* OR activity tracker OR activity monitor OR fitbit*) |
| #3 | TI (randomized controlled trial or randomized or placebo or RCT) OR AB (randomized controlled trial or randomized or placebo or RCT) |
| #4 | #1 AND #2 AND #3 |
