# Supplementary material for: Behavior Change Resources Used in Mobile App–Based Interventions Addressing Weight, Behavioral, and Metabolic Outcomes in Adults With Overweight and Obesity: Systematic Review and Meta-Analysis of Randomized Controlled Trials
Source: JMIR Mhealth Uhealth. 2025 Aug 19;13:e63313. doi: 10.2196/63313 (PMC12392691; doi:10.2196/63313)
Supplement: Multimedia Appendix 3 [file mhealth-v13-e63313-s003.docx]

| Table S3 Studies excluded at full-text review | | |
| --- | --- | --- |
| Number | Reference | Reason |
| 1 | Zhang N, Zhou M, Li M, Ma G. Effects of Smartphone-Based Remote Interventions on Dietary Intake, Physical Activity, Weight Control, and Related Health Benefits Among the Older Population With Overweight and Obesity in China: Randomized Controlled Trial. J Med Internet Res. 2023 Apr 28;25:e41926. PMID: 37115608. doi: 10.2196/41926. | Wrong Population |
| 2 | Yoshimura E, Tajiri E, Michiwaki R, Matsumoto N, Hatamoto Y, Tanaka S. Long-term Effects of the Use of a Step Count-Specific Smartphone App on Physical Activity and Weight Loss: Randomized Controlled Clinical Trial. JMIR Mhealth Uhealth. 2022 Oct 24;10(10):e35628. PMID: 36279159. doi: 10.2196/35628. | Wrong Population |
| 3 | Weinhold KR, Miller CK, Marrero DG, Nagaraja HN, Focht BC, Gascon GM. A Randomized Controlled Trial Translating the Diabetes Prevention Program to a University Worksite, Ohio, 2012-2014. Prev Chronic Dis. 2015 Nov 25;12:E210. PMID: 26605710. doi: 10.5888/pcd12.150301. | Wrong Population |
| 4 | Wang JB, Cataldo JK, Ayala GX, Natarajan L, Cadmus-Bertram LA, White MM, et al. Mobile and Wearable Device Features that Matter in Promoting Physical Activity. J Mob Technol Med. 2016 Jul;5(2):2-11. PMID: 27493694. doi: 10.7309/jmtm.5.2.2. | Wrong Intervention |
| 5 | Wang J. A wearable sensor (fitbit one) and text-messaging to promote physical activity and participants' level of engagement (a randomized controlled feasibility trial): ProQuest Information & Learning; 2015. | Wrong Intervention |
| 6 | Turner-McGrievy GM, Wilcox S, Boutté A, Hutto BE, Singletary C, Muth ER, et al. The Dietary Intervention to Enhance Tracking with Mobile Devices (DIET Mobile) Study: A 6-Month Randomized Weight Loss Trial. Obesity (Silver Spring). 2017 Aug;25(8):1336-42. PMID: 28600833. doi: 10.1002/oby.21889. | Wrong Intervention |
| 7 | Turner-McGrievy GM, Tate DF. Weight loss social support in 140 characters or less: use of an online social network in a remotely delivered weight loss intervention. Translational Behavioral Medicine. 2013 Sep;3(3):287-94. PMID: WOS:000209412900011. doi: 10.1007/s13142-012-0183-y. | Wrong Outcome |
| 8 | Turner-McGrievy G, Tate D. Tweets, Apps, and Pods: Results of the 6-month Mobile Pounds Off Digitally (Mobile POD) randomized weight-loss intervention among adults. Journal of medical Internet research. 2011;13(4):e120. doi: 10.2196/jmir.1841. | Wrong Control |
| 9 | Thorgeirsson T, Torfadottir JE, Egilsson E, Oddsson S, Gunnarsdottir T, Aspelund T, et al. Randomized Trial for Weight Loss Using a Digital Therapeutic Application. Journal of Diabetes Science and Technology. 2022 Sep;16(5):1150-8. PMID: WOS:000898115500013. doi: 10.1177/19322968211000815. | Wrong Intervention |
| 10 | Thomas JG, Bond DS, Raynor HA, Papandonatos GD, Wing RR. Comparison of Smartphone-Based Behavioral Obesity Treatment With Gold Standard Group Treatment and Control: A Randomized Trial. Obesity (Silver Spring). 2019 Apr;27(4):572-80. PMID: 30779333. doi: 10.1002/oby.22410. | Wrong Intervention |
| 11 | Tanaka K, Sasai H, Wakaba K, Murakami S, Ueda M, Yamagata F, et al. Professional dietary coaching within a group chat using a smartphone application for weight loss: a randomized controlled trial. J Multidiscip Healthc. 2018;11:339-47. PMID: 30038502. doi: 10.2147/jmdh.S165422. | Wrong Population |
| 12 | Svetkey LP, Batch BC, Lin PH, Intille SS, Corsino L, Tyson CC, et al. Cell phone intervention for you (CITY): A randomized, controlled trial of behavioral weight loss intervention for young adults using mobile technology. Obesity (Silver Spring). 2015 Nov;23(11):2133-41. PMID: 26530929. doi: 10.1002/oby.21226. | Wrong Population |
| 13 | Suen L, Wang W, Cheng KKY, Chua MCH, Yeung JWF, Koh WK, et al. Self-Administered Auricular Acupressure Integrated With a Smartphone App for Weight Reduction: Randomized Feasibility Trial. JMIR Mhealth Uhealth. 2019 May 29;7(5):e14386. PMID: 31144666. doi: 10.2196/14386. | Unavailable Data |
| 14 | Stephens JD, Yager AM, Allen J. Smartphone Technology and Text Messaging for Weight Loss in Young Adults: a Randomized Controlled Trial. Journal of cardiovascular nursing. 2017;32(1):39‐46. PMID: CN-01455449. doi: 10.1097/JCN.0000000000000307. | Wrong Intervention |
| 15 | Stansbury ML, Krukowski RA, You W, Harvey JR, West DS. Effects of meeting steps-based and minutes-based physical activity goals on weight loss in online behavioral weight control: seemingly unrelated regrassion analysis. Health Psychology and Behavioral Medicine. 2022 Dec;10(1):956-72. PMID: WOS:000863510500001. doi: 10.1080/21642850.2022.2129654. | Wrong Study Design |
| 16 | Spring B, Duncan JM, Janke EA, Kozak AT, McFadden HG, DeMott A, et al. Integrating technology into standard weight loss treatment: a randomized controlled trial. JAMA Intern Med. 2013 Jan 28;173(2):105-11. PMID: 23229890. doi: 10.1001/jamainternmed.2013.1221. | Wrong Intervention |
| 17 | Shuger SL, Barry VW, Sui X, McClain A, Hand GA, Wilcox S, et al. Electronic feedback in a diet- and physical activity-based lifestyle intervention for weight loss: a randomized controlled trial. International journal of behavioral nutrition and physical activity. 2011;8:41. PMID: CN-00799921. doi: 10.1186/1479-5868-8-41. | Wrong Intervention |
| 18 | Seo EY, Kim YS, Lee YJ, Hur MH. Virtual Reality Exercise Program Effects on Body Mass Index, Depression, Exercise Fun and Exercise Immersion in Overweight Middle-Aged Women: A Randomized Controlled Trial. International Journal of Environmental Research and Public Health. 2023;20(2). doi: 10.3390/ijerph20020900. | Wrong Population |
| 19 | Salas-Salvadó J, Díaz-López A, Ruiz-Canela M, Basora J, Fitó M, Corella D, et al. Effect of a Lifestyle Intervention Program With Energy-Restricted Mediterranean Diet and Exercise on Weight Loss and Cardiovascular Risk Factors: One-Year Results of the PREDIMED-Plus Trial. Diabetes Care. 2019 May;42(5):777-88. PMID: 30389673. doi: 10.2337/dc18-0836. | Wrong Population |
| 20 | Roth L, Ordnung M, Forkmann K, Mehl N, Horstmann A. A randomized‐controlled trial to evaluate the app‐based multimodal weight loss program zanadio for patients with obesity. Obesity (19307381). 2023;31(5):1300-10. PMID: 163336919. Language: English. Entry Date: In Process. Revision Date: 20230502. Publication Type: Journal Article. Journal Subset: Biomedical. doi: 10.1002/oby.23744. | Unavailable Data |
| 21 | Roth L, Ordnung M, Forkmann K, Mehl N, Horstmann A. A randomized-controlled trial to evaluate the app-based multimodal weight loss program zanadio for patients with obesity. Obesity (Silver Spring). 2023 May;31(5):1300-10. PMID: 37140392. doi: 10.1002/oby.23744. | Duplicate |
| 22 | Rogers RJ. The comparison of a technology-based system and in-person behavioral weight loss intervention in the severely obese: ProQuest Information & Learning; 2014. | Wrong Intervention |
| 23 | Reading JM, Crane MM, Carlyle K, Perera RA, LaRose JG. A Self-Guided Lifestyle Intervention for Young Men: Findings from the ACTIVATE Randomized Pilot Trial. Journal of Mens Health. 2022 Sep;18(9). PMID: WOS:000890642400014. doi: 10.31083/j.jomh1809191. | Wrong Intervention |
| 24 | Raynor HA, Thomas JG, Cardoso CC, Wojtanowski AC, Foster GD. Examining the pattern of new foods and beverages consumed during obesity treatment to inform strategies for self-monitoring intake. Appetite. 2019;132:147‐53. PMID: CN-01649806. doi: 10.1016/j.appet.2018.10.018. | Wrong Outcome |
| 25 | Pellegrini CA, Conroy DE, Phillips SM, Pfammatter AF, McFadden HG, Spring B. Daily and Seasonal Influences on Dietary Self-monitoring Using a Smartphone Application. J Nutr Educ Behav. 2018 Jan;50(1):56-61.e1. PMID: 29325663. doi: 10.1016/j.jneb.2016.12.004. | Wrong Outcome |
| 26 | Patel MS, Small DS, Harrison JD, Fortunato MP, Oon AL, Rareshide CAL, et al. Effectiveness of Behaviorally Designed Gamification Interventions With Social Incentives for Increasing Physical Activity Among Overweight and Obese Adults Across the United States The STEP UP Randomized Clinical Trial. Jama Internal Medicine. 2019 Dec;179(12):1624-32. PMID: WOS:000505189200004. doi: 10.1001/jamainternmed.2019.3505. | Wrong Intervention |
| 27 | Patel ML, Hopkins CM, Brooks TL, Bennett GG. Comparing Self-Monitoring Strategies for Weight Loss in a Smartphone App: randomized Controlled Trial. JMIR mHealth and uHealth. 2019;7(2):e12209. PMID: CN-02078271. doi: 10.2196/12209. | Wrong Control |
| 28 | Patel ML, Hopkins CM, Bennett GG. Early weight loss in a standalone mHealth intervention predicting treatment success. Obesity Science and Practice. 2019;5(3):231-7. doi: 10.1002/osp4.329. | Wrong Control |
| 29 | Patel ML. Comparing self-monitoring strategies for weight loss: Does developing mastery before diet tracking enhance engagement? : ProQuest Information & Learning; 2019. | Wrong Control |
| 30 | Pagoto SL, Schroeder MW, Xu R, Waring ME, Groshon L, Goetz JM, et al. A Facebook-Delivered Weight Loss Intervention Using Open Enrollment: Randomized Pilot Feasibility Trial. Jmir Formative Research. 2022 May;6(5). PMID: WOS:000854077500019. doi: 10.2196/33663. | Wrong Control |
| 31 | Oh B, Yi GH, Han MK, Kim JS, Lee CH, Cho B, et al. Importance of Active Participation in Obesity Management Through Mobile Health Care Programs: Substudy of a Randomized Controlled Trial. JMIR Mhealth Uhealth. 2018 Jan 3;6(1):e2. PMID: 29298749. doi: 10.2196/mhealth.8719. | Wrong Population |
| 32 | Nezami BT, Hurley L, Power J, Valle CG, Tate DF. A pilot randomized trial of simplified versus standard calorie dietary self-monitoring in a mobile weight loss intervention. Obesity (Silver Spring). 2022 Mar;30(3):628-38. PMID: 35146942. doi: 10.1002/oby.23377. | Wrong Control |
| 33 | Nanri A, Tomita K, Matsushita Y, Ichikawa F, Yamamoto M, Nagafuchi Y, et al. Effect of six months lifestyle intervention in Japanese men with metabolic syndrome: randomized controlled trial. J Occup Health. 2012;54(3):215-22. PMID: 22790524. doi: 10.1539/joh.11-0238-oa. | Wrong Population |
| 34 | Mummah SA, Mathur M, King AC, Gardner CD, Sutton S. Mobile Technology for Vegetable Consumption: A Randomized Controlled Pilot Study in Overweight Adults. JMIR mhealth and uhealth. 2016;4(2):e51. PMID: CN-02547911. doi: 10.2196/mhealth.5146. | Wrong Outcome |
| 35 | Mummah S, Robinson TN, Mathur M, Farzinkhou S, Sutton S, Gardner CD. Effect of a mobile app intervention on vegetable consumption in overweight adults: a randomized controlled trial. Int J Behav Nutr Phys Act. 2017 Sep 15;14(1):125. PMID: 28915825. doi: 10.1186/s12966-017-0563-2. | Wrong Outcome |
| 36 | Moss J, Tew GA, Copeland RJ, Stout M, Billings CG, Saxton JM, et al. Effects of a pragmatic lifestyle intervention for reducing body mass in obese adults with obstructive sleep apnoea: a randomised controlled trial. Biomed Res Int. 2014;2014:102164. PMID: 25136550. doi: 10.1155/2014/102164. | Wrong Population |
| 37 | Monroe CM, Geraci M, Larsen CA, West DS. Feasibility and efficacy of a novel technology-based approach to harness social networks for weight loss: the NETworks pilot randomized controlled trial. Obes Sci Pract. 2019 Aug;5(4):354-65. PMID: 31452920. doi: 10.1002/osp4.352. | Wrong Control |
| 38 | Miller NA, Ehmann MM, Hagerman CJ, Forman EM, Arigo D, Spring B, et al. Sharing digital self-monitoring data with others to enhance long-term weight loss: a randomized controlled trial. Contemporary clinical trials. 2023;129:107201. PMID: CN-02553726. doi: 10.1016/j.cct.2023.107201. | Wrong Control |
| 39 | Merchant G, Weibel N, Patrick K, Fowler JH, Norman GJ, Gupta A, et al. Click "like" to change your behavior: a mixed methods study of college students' exposure to and engagement with Facebook content designed for weight loss. J Med Internet Res. 2014 Jun 24;16(6):e158. PMID: 24964294. doi: 10.2196/jmir.3267. | Wrong Outcome |
| 40 | Mateo KF. A mobile health app for weight loss that incorporates social networking. Journal of clinical outcomes management. 2016;23(11):498‐500. PMID: CN-01291895. | Wrong Control |
| 41 | Martineta M, Agustina R, Febriyanti E, Putri M, Purnamasari D. A balanced-sustainable calorie-restricted diet effect using “eats up”application on waist circumference and inflammatory marker among Indonesian obese women: Randomized clinical trial. Annals of Nutrition and Metabolism. 2019;75(3):62. doi: 10.1159/000501751. | Conference Abstract |
| 42 | Martin CK, Miller AC, Thomas DM, Champagne CM, Han H, Church T. Efficacy of SmartLoss, a smartphone-based weight loss intervention: results from a randomized controlled trial. Obesity (Silver Spring). 2015 May;23(5):935-42. PMID: 25919921. doi: 10.1002/oby.21063. | Wrong Intervention |
| 43 | Martin CK, Miller AC, Thomas DM, Champagne CM, Han H, Church T. Efficacy of SmartLoss<sup>SM</sup>, a smartphone-based weight loss intervention: Results from a randomized controlled trial. Obesity. 2015;23(5):935-42. doi: 10.1002/oby.21063. | Duplicate |
| 44 | Lyons EJ, Swartz MC, Lewis ZH, Martinez E, Jennings K. Feasibility and Acceptability of a Wearable Technology Physical Activity Intervention With Telephone Counseling for Mid-Aged and Older Adults: A Randomized Controlled Pilot Trial. JMIR Mhealth Uhealth. 2017 Mar 6;5(3):e28. PMID: 28264796. doi: 10.2196/mhealth.6967. | Wrong Population |
| 45 | López D, Torres M, Vélez J, Grullon J, Negrón E, Pérez CM, et al. Development and Evaluation of a Nutritional Smartphone Application for Making Smart and Healthy Choices in Grocery Shopping. Healthc Inform Res. 2017 Jan;23(1):16-24. PMID: 28261527. doi: 10.4258/hir.2017.23.1.16. | Wrong Outcome |
| 46 | Lewis ZH, Ottenbacher KJ, Fisher SR, Jennings K, Brown AF, Swartz MC, et al. Effect of electronic activity monitors and pedometers on health: Results from the tame health pilot randomized pragmatic trial. International Journal of Environmental Research and Public Health. 2020;17(18):1-11. doi: 10.3390/ijerph17186800. | Wrong Intervention |
| 47 | Larsen RN, Mann NJ, Maclean E, Shaw JE. The effect of high-protein, low-carbohydrate diets in the treatment of type 2 diabetes: a 12 month randomised controlled trial. Diabetologia. 2011 Apr;54(4):731-40. PMID: 21246185. doi: 10.1007/s00125-010-2027-y. | Wrong Population |
| 48 | Lin M, Mahmooth Z, Dedhia N, Frutchey R, Mercado CE, Epstein DH, et al. Tailored, interactive text messages for enhancing weight loss among African American adults: the TRIMM randomized controlled trial. Am J Med. 2015 Aug;128(8):896-904. PMID: 25840035. doi: 10.1016/j.amjmed.2015.03.013. | Wrong Intervention |
| 49 | Laing BY, Mangione CM, Tseng CH, Leng M, Vaisberg E, Mahida M, et al. Effectiveness of a smartphone application for weight loss compared with usual care in overweight primary care patients: a randomized, controlled trial. Ann Intern Med. 2014 Nov 18;161(10 Suppl):S5-12. PMID: 25402403. doi: 10.7326/m13-3005. | Wrong Control |
| 50 | Kitazawa M, Takeda Y, Hatta M, Horikawa C, Sato T, Osawa T, et al. "Lifestyle Intervention with Smartphone app and isCGM for People at High Risk of Type 2 Diabetes: Randomized Trial". J Clin Endocrinol Metab. 2023 Nov 1. PMID: 37931069. doi: 10.1210/clinem/dgad639. | Wrong Population |
| 51 | Kim M, Kim Y, Go Y, Lee S, Na M, Lee Y, et al. Multidimensional Cognitive Behavioral Therapy for Obesity Applied by Psychologists Using a Digital Platform: open-Label Randomized Controlled Trial. JMIR mHealth and uHealth. 2020;8(4):e14817. PMID: CN-02121890. doi: 10.2196/14817. | Wrong Control |
| 52 | Kempf K, Röhling M, Martin S, Schneider M. Telemedical coaching for weight loss in overweight employees: a three-armed randomised controlled trial. BMJ Open. 2019 Apr 11;9(4):e022242. PMID: 30975666. doi: 10.1136/bmjopen-2018-022242. | Wrong Intervention |
| 53 | Kariuki JK, Bizhanova Z, Conroy MB, Burke LE, Cheng J, Beatrice B, et al. The Association between Neighborhood Walkability and Physical Activity in a Behavioral Weight Loss Trial Testing the Addition of Remotely Delivered Feedback Messages to Self-Monitoring. Behavioral Medicine. 2023. doi: 10.1080/08964289.2023.2238102. | Wrong Intervention |
| 54 | Kakoschke N, Hawker C, Castine B, De Courten B, Verdejo-Garcia A. Modification of cognitive biases in overweight and obesity. Obesity research & clinical practice. 2019;13(3):268. PMID: CN-01962560. doi: 10.1016/j.orcp.2018.11.094. | Wrong Control |
| 55 | Kakoschke N, Hawker C, Castine B, de Courten B, Verdejo-Garcia A. Smartphone-based cognitive bias modification training improves healthy food choice in obesity: A pilot study. Eur Eat Disord Rev. 2018 Sep;26(5):526-32. PMID: 30003634. doi: 10.1002/erv.2622. | Wrong Control |
| 56 | Júdice PB, Santos DA, Hamilton MT, Sardinha LB, Silva AM. Validity of GT3X and Actiheart to estimate sedentary time and breaks using ActivPAL as the reference in free-living conditions. Gait & Posture. 2015 May;41(4):917-22. PMID: WOS:000353401000009. doi: 10.1016/j.gaitpost.2015.03.326. | Wrong Intervention |
| 57 | Joseph RP, Todd M, Ainsworth BE, Vega-López S, Adams MA, Hollingshead K, et al. Smart Walk: A Culturally Tailored Smartphone-Delivered Physical Activity Intervention for Cardiometabolic Risk Reduction among African American Women. International Journal of Environmental Research and Public Health. 2023;20(2). doi: 10.3390/ijerph20021000. | Wrong Control |
| 58 | Johnston CA, Rost S, Miller-Kovach K, Moreno JP, Foreyt JP. A randomized controlled trial of a community-based behavioral counseling program. Am J Med. 2013 Dec;126(12):1143.e19-24. PMID: 24135513. doi: 10.1016/j.amjmed.2013.04.025. | Unavailable Data |
| 59 | Johnston CA, Rost S, Miller-Kovach K, Moreno JP, Foreyt JP. A Randomized Controlled Trial of a Community-based Behavioral Counseling Program. American Journal of Medicine. 2013 Dec;126(12). PMID: WOS:000327393300052. doi: 10.1016/j.amjmed.2013.04.025. | Duplicate |
| 60 | Jakicic JM, Davis KK, Rogers RJ, King WC, Marcus MD, Helsel D, et al. Effect of Wearable Technology Combined With a Lifestyle Intervention on Long-term Weight Loss: The IDEA Randomized Clinical Trial. Jama. 2016 Sep 20;316(11):1161-71. PMID: 27654602. doi: 10.1001/jama.2016.12858. | Wrong Intervention |
| 61 | Ipjian ML, Johnston CS. Smartphone technology facilitates dietary change in healthy adults. Nutrition. 2017 Jan;33:343-7. PMID: 27742102. doi: 10.1016/j.nut.2016.08.003. | Wrong Population |
| 62 | Hernández-Reyes A, Cámara-Martos F, Molina-Luque R, Moreno-Rojas R. Effect of an mHealth Intervention Using a Pedometer App With Full In-Person Counseling on Body Composition of Overweight Adults: Randomized Controlled Weight Loss Trial. JMIR Mhealth Uhealth. 2020 May 27;8(5):e16999. PMID: 32348263. doi: 10.2196/16999. | Wrong Intervention |
| 63 | Harvie M, Wright C, Pegington M, McMullan D, Mitchell E, Martin B, et al. The effect of intermittent energy and carbohydrate restriction v. daily energy restriction on weight loss and metabolic disease risk markers in overweight women. Br J Nutr. 2013 Oct;110(8):1534-47. PMID: 23591120. doi: 10.1017/s0007114513000792. | Wrong Intervention |
| 64 | Hales SB. Refinement and pilot testing social networks for encouraging healthy behaviors: The social pounds off digitally (social POD) study: ProQuest Information & Learning; 2016. | Wrong Control |
| 65 | Hales S, Turner-McGrievy GM, Wilcox S, Fahim A, Davis RE, Huhns M, et al. Social networks for improving healthy weight loss behaviors for overweight and obese adults: A randomized clinical trial of the social pounds off digitally (Social POD) mobile app. Int J Med Inform. 2016 Oct;94:81-90. PMID: 27573315. doi: 10.1016/j.ijmedinf.2016.07.003. | Wrong Control |
| 66 | Grossman JA, Arigo D, Bachman JL. Meaningful weight loss in obese postmenopausal women: a pilot study of high-intensity interval training and wearable technology. Menopause. 2018 Apr;25(4):465-70. PMID: 29088015. doi: 10.1097/gme.0000000000001013. | Wrong Intervention |
| 67 | Griffith DM, Pennings JS, Jaeger EC. Mighty Men: A Pilot Test of the Feasibility and Acceptability of a Faith-Based, Individually Tailored, Cluster-Randomized Weight Loss Trial for Middle-Aged and Older African American Men. Am J Mens Health. 2023 Jul-Aug;17(4):15579883231193235. PMID: 37608590. doi: 10.1177/15579883231193235. | Wrong Intervention |
| 68 | Gorin AA, Raynor HA, Fava J, Maguire K, Robichaud E, Trautvetter J, et al. Randomized controlled trial of a comprehensive home environment-focused weight-loss program for adults. Health Psychol. 2013 Feb;32(2):128-37. PMID: 22309885. doi: 10.1037/a0026959. | Wrong Intervention |
| 69 | Gorin AA, Powers TA, Gettens K, Cornelius T, Koestner R, Mobley AR, et al. A randomized controlled trial of a theory-based weight-loss program for couples. Health Psychol. 2020 Feb;39(2):137-46. PMID: 31789558. doi: 10.1037/hea0000808. | Wrong Intervention |
| 70 | Goldstein SP, Goldstein CM, Bond DS, Raynor HA, Wing RR, Thomas JG. Associations Between Self-Monitoring and Weight Change in Behavioral Weight Loss Interventions. Health Psychology. 2019;38(12):1128-36. doi: 10.1037/hea0000800. | Wrong Intervention |
| 71 | Fukuoka Y, Vittinghoff E, Hooper J. A weight loss intervention using a commercial mobile application in Latino Americans-Adelgaza Trial. Transl Behav Med. 2018 Sep 8;8(5):714-23. PMID: 29474702. doi: 10.1093/tbm/ibx039. | Wrong Study Design |
| 72 | Forman EM, Goldstein SP, Crochiere RJ, Butryn ML, Juarascio AS, Zhang F, et al. Randomized controlled trial of OnTrack, a just-in-time adaptive intervention designed to enhance weight loss. Transl Behav Med. 2019 Nov 25;9(6):989-1001. PMID: 31602471. doi: 10.1093/tbm/ibz137. | Wrong Control |
| 73 | Fenton S, Burrows TL, Collins CE, Rayward AT, Murawski B, Duncan MJ. Efficacy of a Multi-Component m-Health Diet, Physical Activity, and Sleep Intervention on Dietary Intake in Adults with Overweight and Obesity: A Randomised Controlled Trial. Nutrients. 2021 Jul 19;13(7). PMID: 34371975. doi: 10.3390/nu13072468. | Unavailable Data |
| 74 | Falkenhain K, Locke SR, Lowe DA, Reitsma NJ, Lee T, Singer J, et al. Keyto app and device versus WW app on weight loss and metabolic risk in adults with overweight or obesity: A randomized trial. Obesity (Silver Spring). 2021 Oct;29(10):1606-14. PMID: 34124856. doi: 10.1002/oby.23242. | Wrong Control |
| 75 | Falkenhain K, Locke SR, Lowe DA, Lee T, Singer J, Weiss EJ, et al. Use of an mHealth Ketogenic Diet App Intervention and User Behaviors Associated With Weight Loss in Adults With Overweight or Obesity: Secondary Analysis of a Randomized Clinical Trial. JMIR Mhealth Uhealth. 2022 Mar 14;10(3):e33940. PMID: 35285809. doi: 10.2196/33940. | Wrong Study Design |
| 76 | Eisenhauer CM, Brito F, Kupzyk K, Yoder A, Almeida F, Beller RJ, et al. Mobile health assisted self-monitoring is acceptable for supporting weight loss in rural men: a pragmatic randomized controlled feasibility trial. BMC Public Health. 2021 Aug 18;21(1):1568. PMID: 34407782. doi: 10.1186/s12889-021-11618-7. | Wrong Control |
| 77 | Dunn CG, Turner-McGrievy GM, Wilcox S, Hutto B. Dietary Self-Monitoring Through Calorie Tracking but Not Through a Digital Photography App Is Associated with Significant Weight Loss: The 2SMART Pilot Study-A 6-Month Randomized Trial. J Acad Nutr Diet. 2019 Sep;119(9):1525-32. PMID: 31155474. doi: 10.1016/j.jand.2019.03.013. | Wrong Control |
| 78 | dos Santos MM, De Marchi ACB, de Leon EB, Roque LG. Health behaviour change and activation in e-health weight loss intervention for elderly: a randomised clinical trial. Behaviour & Information Technology. 2023 2023 Jun. PMID: WOS:001003538100001. doi: 10.1080/0144929x.2023.2222181. | Unavailable Data |
| 79 | Cheng J, Costacou T, Sereika SM, Conroy MB, Parmanto B, Rockette-Wagner B, et al. Effect of an mHealth weight loss intervention on Healthy Eating Index diet quality: the SMARTER randomised controlled trial. British Journal of Nutrition. 2023 2023 Jun. PMID: WOS:001017789800001. doi: 10.1017/s0007114523001137. | Wrong Outcome |
| 80 | Cavallo DN, Martinez R, Webb Hooper M, Flocke S. Feasibility of a social media-based weight loss intervention designed for low-SES adults. Transl Behav Med. 2021 Apr 26;11(4):981-92. PMID: 32716040. doi: 10.1093/tbm/ibaa070. | Wrong Control |
| 81 | Carter MC, Burley VJ, Cade JE. Weight Loss Associated With Different Patterns of Self-Monitoring Using the Mobile Phone App My Meal Mate. Jmir Mhealth and Uhealth. 2017 Feb;5(2). PMID: WOS:000395837000007. doi: 10.2196/mhealth.4520. | Unavailable Data |
| 82 | Carter MC, Burley VJ, Cade JE. Results of a pilot trial of “My Meal Mate” (MMM), a new smartphone application designed to facilitate weight loss. Obesity facts. 2013;6:40. PMID: CN-01065118. | Unavailable Data |
| 83 | Carpenter CA, Eastman A, Ross KM. Consistency With and Disengagement From Self-monitoring of Weight, Dietary Intake, and Physical Activity in a Technology-Based Weight Loss Program: Exploratory Study. Jmir Formative Research. 2022 Feb;6(2). PMID: WOS:000854070800028. doi: 10.2196/33603. | Wrong Intervention |
| 84 | Cadmus-Bertram LA, Marcus BH, Patterson RE, Parker BA, Morey BL. Randomized Trial of a Fitbit-Based Physical Activity Intervention for Women. American Journal of Preventive Medicine. 2015;49(3):414-8. doi: 10.1016/j.amepre.2015.01.020. | Wrong Intervention |
| 85 | Cadmus-Bertram L, Marcus BH, Patterson RE, Parker BA, Morey BL. Use of the Fitbit to Measure Adherence to a Physical Activity Intervention Among Overweight or Obese, Postmenopausal Women: Self-Monitoring Trajectory During 16 Weeks. JMIR Mhealth Uhealth. 2015 Nov 19;3(4):e96. PMID: 26586418. doi: 10.2196/mhealth.4229. | Wrong Intervention |
| 86 | Burke LE, Zheng Y, Ma Q, Mancino J, Loar I, Music E, et al. The SMARTER pilot study: Testing feasibility of real-time feedback for dietary self-monitoring. Preventive Medicine Reports. 2017;6:278-85. doi: 10.1016/j.pmedr.2017.03.017. | Wrong Intervention |
| 87 | Burke LE, Styn MA, Sereika SM, Conroy MB, Ye L, Glanz K, et al. Using mHealth technology to enhance self-monitoring for weight loss: a randomized trial. American Journal of Preventive Medicine. 2012;43(1):20-6. PMID: 104463276. Language: English. Entry Date: 20121026. Revision Date: 20211029. Publication Type: Journal Article. doi: 10.1016/j.amepre.2012.03.016. | Wrong Intervention |
| 88 | Burke LE, Sereika SM, Parmanto B, Bizhanova Z, Kariuki JK, Cheng J, et al. Effect of tailored, daily feedback with lifestyle self-monitoring on weight loss: The SMARTER randomized clinical trial. Obesity. 2022;30(1):75-84. doi: 10.1002/oby.23321. | Wrong Intervention |
| 89 | Burke LE, Sereika SM, Bizhanova Z, Parmanto B, Kariuki J, Cheng J, et al. The Effect of Tailored, Daily, Smartphone Feedback to Lifestyle Self-Monitoring on Weight Loss at 12 Months: the SMARTER Randomized Clinical Trial. Journal of Medical Internet Research. 2022;24(7). doi: 10.2196/38243. | Duplicate |
| 90 | Brindal E, Freyne J, Saunders I, Berkovsky S, Smith G, Noakes M. Features predicting weight loss in overweight or obese participants in a web-based intervention: randomized trial. J Med Internet Res. 2012 Dec 12;14(6):e173. PMID: 23234759. doi: 10.2196/jmir.2156. | Wrong Intervention |
| 91 | Alick CL, Samuel-Hodge C, Ward D, Ammerman A, Rini C, Tate DF. Together Eating & Activity Matters (TEAM): results of a pilot randomized-clinical trial of a spousal support weight loss intervention for Black men. Obes Sci Pract. 2018 Feb;4(1):62-75. PMID: 29479466. doi: 10.1002/osp4.142. | Wrong Intervention |
