# Supplementary material for: Behavior Change Resources Used in Mobile App–Based Interventions Addressing Weight, Behavioral, and Metabolic Outcomes in Adults With Overweight and Obesity: Systematic Review and Meta-Analysis of Randomized Controlled Trials
Source: JMIR Mhealth Uhealth. 2025 Aug 19;13:e63313. doi: 10.2196/63313 (PMC12392691; doi:10.2196/63313)
Supplement: Multimedia Appendix 4 [file mhealth-v13-e63313-s004.docx]

| Table S4 Characteristics of included studies | | | | | | | | | |
| --- | --- | --- | --- | --- | --- | --- | --- | --- | --- |
| Author (Reference) | Year/Location | Sample Size (IG/CG) | Gender (Male/Female) | Mean Age  (IG/CG) | Mean BMI (IG/CG) | Intervention | Comparison | Duration (Months) | Main Results |
| Apiñaniz, A. [1] | 2019/Spain | Baseline: 110 (54/56); follow-up: 66 (33/33) | IG: 13/41; CG: 18/38 | 38.8±5.4/38.3±4.5 | 33.4±5.3/32.1±4.5 | Advice regarding physical activity and diet was reinforced using an app AKTIDIET®. | Motivational advice including recommendations on diet and physical exercise. | 6 | - Weight reduction (0.357 kg, *P*= 0.7); - HbA1c differences (−0.095%, *P*= 0.046). |
| Bughin, F. [2] | 2021/France | Baseline: 50 (25/25); follow-up: 49 (24/25) | IG: 11/33; CG: 12/36 | 51.2±10.8/53.3±11.3 | 36.2±4.2/36.82±5.0 | Telemouv app was installed, and contains 3 components: PA, nutritional, and educational programs. | Sessions focusing on endurance PA sessions and received a booklet with different exercises and tips on PA and nutrition management. | 3 | - Weight changed from 105.72 kg to 105.26 (intervention) and 104.41 to 104.89 (control, *P*= 0.89); - BMI changed from 36.22 kg/m^2^ to 36.02 (intervention) and 36.82 to 36.98 (control, *P*= 0.54); - Fat mass changed from 44.80 kg to 43.18 (intervention) and 43.56 to 43.18 (control, *P*= 0.94). |
| Carter, M. C. [3] | 2013/UK | Baseline: 128 (43/43/42); follow-up: 79 (40/19/20) | IG: 10/33; CG1: 10/32; CG2: 9/34 | IG: 10/33; CG1: 10/32; CG2: 9/34 | 33.7±4.2/34.5±5.6/34.5±5.7 | The MMM app allows users to set a weight loss goal and self-monitor daily calorie intake toward achieving that goal. Physical activity can also be recorded in the diary. | The comparison groups used either a self-monitoring slimming website or a food diary and provided an opportunity to deliver a similar self-monitoring intervention by different mediums. | 6 | - Weight changed from 96.8 kg to 92.2 (IG1), 96.4 to 95.1 (IG2) and 97.9 to 95.0 (CG, *P<* 0.001); - BMI changed from 33.7 kg/m^2^ to 32.1 (IG1), 34.5 to 34.0 (IG2), and 34.5 to 33.4 (CG, *P<* 0.001). |
| Choi, J. H. [4] | 2023/Korea | Baseline: 30 (15/15); follow-up: 30 (15/15) | IG: 0/15; CG: 0/15 | 39.70±10.07/39.20±11.63 | NA | The EXP was performed non-face-to-face using an m-health exercise intervention. | The CON group was encouraged to maintain their normal daily routines. | 3 | - Weight reduction -0.85 kg in IG, and -0.17 in CG; - BMI reduction -0.29 kg/m^2^ in IG, and -0.09 in CG; - Fat mass reduction -1.47 kg in IG (*P*<0.001), and 0.55 in CG; - SBP reduction -2.13 mmHg in IG, and -0.34 in CG; - DBP reduction -3.59 mmHg in IG, and -0.33 in CG. |
| Domal, S. V. [5] | 2023/India | Baseline: 20 (10/10); follow-up: 19 (9/10) | IG: 7/3; CG: 6/4 | 24±3/27±11 | 27.40±2.60/28.40±0.65 | The EXi app creates a digital personalized physical exercise plan for the next 12 weeks. | Participants continued their routine daily work. | 2 | - Weight increase 0.16 kg in IG, and 0.71 in CG (*P*=0.09); - BMI increase 0.03 kg/m^2^ in IG, and 0.3 in CG (*P*=0.33); - WC reduction -1.20 cm in IG, and -0.70 in CG (*P*=0.07); - Fat mass reduction -0.33 kg in IG, and -0.37 in CG (*P*=0.06); - SBP increase 2.50 mmHg in IG, and 5.50 in CG (*P*=0.81); - DBP increase 0.33 mmHg in IG, and 3.5 in CG (*P*=0.75). |
| Duncan, M. J. [6] | 2020/Australia | Baseline: 116 (39/41/36);  6-month follow-up: 80 (27/32/21);  12-month follow-up: 54 (14/23/17) | IG1: 12/27; IG2: 11/30; CG: 11/25 | 47.2±9.4/45.4±10.2/40.5±10.7 | 31.9±4.0/31.4±3.8/31.9±3.9 | Balanced app was used to set goals and self-monitor weight and target behaviors and received dynamic feedback on performance. The Enhanced group also received access to a sleep intervention via the app and participant handbook. | No intervention. | 6 | - Weight difference -0.92 kg (95% CI -3.33 to 1.48) between IG and CG; - WC differnce -0.62 cm (95% CI -3.18 to 1.95) between IG and CG; - MVPA difference 2.63 min/day (95% CI -12.22 to 17.48) between IG and CG; - Energy uintake difference -1037.03 kJ/day (95% CI -2028.84 to -45.22) between IG and CG; - HbA1c difference 0.01 (95% CI -0.15 to 0.16) between IG and CG. |
| Godino, J. G. [7] | 2016/USA | Baseline: 404 (202/202); 6-month follow-up: 185/196; 12-month follow-up: 184/193; 18-month follow-up: 164/183; 24-month follow-up: 162/179 | IG: 72/71; CG: 71/70 | NA | 28.9±2.8/29.0±2.7 | The SMART intervention was remotely delivered via six modalities, Intervention participants were instructed to use at least one or more modality a minimum of five times per week throughout the 24 months of intervention. | Participants were given access to a different website than intervention participants and were sent quarterly newsletters via email. | 24 | - Weight difference -0.79 kg (95% CI -2.02 to 0.43) between IG and CG; - BMI difference -0.28 kg/m^2^ (95% CI -0.71 to 0.15) between IG and CG; - WC difference -0.98 cm (95% CI -2.06 to 0.96) between IG and CG; - SBP difference 2.09 mmHg (95% CI 0.21 to 3.97) between IG and CG; - DBP difference 0.85 mmHg (95% CI -0.59 to 2.29) between IG and CG. |
| Hebden, L. [8] | 2014/Australia | Baseline: 51 (26/25); follow-up: 46 (21/25) | IG: 22/4; CG: 19/6 | 22.6±5.4/23.1±3.7 | NA | This program addressed four key lifestyle behavior associated with weight gain during young adulthood: physical activity and sedentary behaviors, intake of fruit and vegetables, energy-dense takeaway meals and sugar sweetened beverages. | Received printed diet booklet with instructions prepared by a dietitian. | 3 | - Weight difference -0.30 kg (95% CI -1.84 to 1.24) between IG and CG; - BMI difference -0.11 kg/m^2^ (95% CI -0.66 to 0.43) between IG and CG; - MVPA difference 1.81 min/day (95% CI -11.17 to 14.80) between IG and CG. |
| Hurkmans, E. [9] | 2018/Belgium | Baseline: 102 (28/30/22/22); follow-up: 81 (21/24/18/18) | Female%  IG1: 84%; IG2: 72%; IG3: 48%; CG: 75% | 46±9.2/44±12.4/45±9.6/45±10.2 | Weight, kg (IG1/IG2/IG3/CG): 90±9.1/90±10.1/96±12/92±10.2 | The app consisted of six parts: digital advice for their dietary pattern and physical activity, how to challenge themselves, self-monitoring, library with information on nutrition and physical activity but also recipes, a help button for advice, and a link to a Facebook group. | Participants were informed that they were on the waiting list for the weight loss program. | 3 | - BMI MD (SD) at post-IG1 -1.0 kg/m^2^ (1.3), post-IG2 -0.7 (1.0), post-IG3 -1.3 (1.2), post-CG 0.1 (1.0); - MVPA MD (SD) at post-IG1 11.8 min/day (61.4), post-IG2 3.6 (72.8), post-IG3 -33.5 (39.8), post-CG -1.7 (63.7); - Energy intake MD (SD) at post-IG1 -392.7 kJ/day (302.9), post-IG2 -192.2 (247.4), post-IG3 -287.3 (277.3), post-CG -115.1 (381.8). |
| Hutchesson, M. J. [10] | 2018/Australia | Baseline: 57 (29/28); follow-up: 42 (22/21) | NA | 26.3±4.3/27.9±5.0 | 29.3±2.5/29.4±2.5 | BPBH is a six-month weight loss program delivered using e-Health technologies only, comprising five delivery modes, including Be Positive Be Healthe Website, self-monitoring app Easy Diet Diary, Email letters and Text messages sent to participants, and a Facebook group. | No intervention. | 6 | - Weight difference -1.94 kg (95% CI -4.31 to 0.42) between IG and CG; - BMI difference -0.68 kg/m^2^ (95% CI -1.47 to 1.09) between IG and CG; - WC difference -1.4 cm (95% CI -3.8 to 1.0) between IG and CG; - Fat mass difference -3.10 kg (95% CI -5.69 to 0.52) between IG and CG; - Energy intake difference -804 kJ/day (95% CI -2524 to 916) between IG and CG; - SBP difference -2.9 mmHg (95% CI -6.8 to 1.0) between IG and CG; - DBP difference -1.9 mmHg (95% CI -5.1 to 1.4) between IG and CG; - Triglycerides difference 0.13 mmol/L (95% CI -0.19 to 0.45) between IG and CG. |
| Jiang, W. Z. [11] | 2021/China | Baseline: 272 (136/136); follow-up: 254 (123/131) | IG: 72/51; CG: 77/54 | 31.52±6.43/32.16±6.39 | 32.36±3.40/32.48±3.59 | Participants in the CIMWM group were provided with two Fit Nutrition Bars per day in the first 3 months as well as monthly face-to-face guidance and daily online instructions via the mobile application “Medical Weight Management”. In the final 3 months, all participants were transferred to the self-monitoring period of the program. | The TMWM group underwent a separate program with monthly face-to-face guidance. In the final 3 months, all participants were transferred to the self-monitoring period of the program. | 6 | - Weight reduction -7.42 kg±8.16 in IG, and -6.86 ±10.67 in CG (*P*=0.46); - BMI reduction -2.44 kg/m^2^±1.82 in IG, and -1.74±2.59 in CG (*P*=0.006); - WC reduction -7.33 cm±8.93 in IG, and -5.70±12.25 in CG (*P*=0.095); - Fat mass reduction -4.62 kg±3.54 in IG, and -3.36±5.67 in CG (*P*=0.002); - SBP reduction -6.66 mmHg±12.97 in IG, and -6.32±13.64 in CG (*P*=0.531); - DBP reduction -5.64 mmHg±10.25 in IG, and -6.14±10.52 in CG (*P*=0.829); - Triglyderides reduction -0.48 mmol/L±1.63 in IG, and -0.30±1.17 in CG (*P*=0.713); - HbA1c reduction -0.04%±0.41 in IG, and -0.02±0.45 in CG (*P*=0.885). |
| Kliemann, N. [12] | 2019/UK | Baseline: 81 (28/27/26); follow-up: 62 (17/19/26) | IG1: 4/24; IG2: 2/25; CG: 2/24 | 43.6±13/44.0±14/40.6±13 | 33.7±7/35.0±8/34.0±7 | The apps advised users to make context-specific plans to turn each tip into a habit and adjust these whenever needed, asked users to track their weight in kg and adherence to the tips each day, and provided automatic updates of how many times each tip was achieved per week as well as daily reminders to promote engagement with the app. | No intervention. | 3 | - Weight reduction -4.50 kg (95% CI -0.93 to 0.27) in IG1, -1.90 (95% CI -4.4 to 0.43) in IG2, and -0.15 (95% CI -5.24 to 4.95) in CG. |
| Lugones-Sanchez, C. [13] | 2022/Spain | Baseline: 650 (318/332); 12-month follow-up: 443 (216/227) | IG: 104/214; CG: 101/231 | 47.7±10.1/48.9±9.2 | 33.1±3.4/33.0±3.6 | The app was designed to allow full daily self-monitoring of food intake and automatically record physical activity through the smart band. | A trained nurse at each primary health center, gave 5 minutes of lifestyle counseling to both groups (CG and IG) before randomization. | 3 | - Weight difference -0.26 kg (95% CI -1.21 to 0.70) between IG and CG; - BMI difference -0.06 kg/m^2^ (95% CI -0.41 to 0.28) between IG and CG; - WC difference -0.48 cm (95% CI -1.62 to 0.66) between IG and CG; - Energy intake -72.40 kcal/day (-202.13 to 57.33) between IG and CG. |
| Lugones-Sanchez, C. [14] | 2020/Spain | Baseline: 440 (231/209); 3-month follow-up: 440 (231/209) | IG: 70/161; CG: 65/144 | 47.4±10.0/48.8±9.2 | 32.8±3.3/32.9±3.4 | The app was designed to allow full daily self-monitoring of food intake and automatically record physical activity through the smart band. | A trained nurse at each primary health center, gave 5 minutes of lifestyle counseling to both groups. | 3 | - Weight difference -0.84 kg (95% CI -1.45 to -0.23) between IG and CG; - BMI difference -0.54 kg/m^2^ (95% CI -0.84 to -0.24) between IG and CG; - Fat mass difference -0.73 kg (95% CI -1.59 to 0.14) between IG and CG. |
| Nakata, Y. [15] | 2022/Japan | Baseline: 141 (72/69); 3-month follow-up: 140 (71/69) | IG: 53/21; CG: 51/23 | 42.3±9.4/44.0±9.1 | 27.3±3.3/27.8±3.7 | Participants were requested to install CALO mama Plus, register their daily weight, diet, exercise, mood, and sleep quality. CALO mama Plus could show their lifestyle changes graphically, evaluate their lifestyle, and indicate some tips for improvement. | No intervention. | 3 | - Weight reduction mean±SD -2.4 kg±4.0 in IG, and -0.7±3.3 in CG (*P*=0.01); - MVPA change mean±SD -2.0 min/day±33.3 in IG, and -7.5±20.2 in CG (*P*=0.11); - Energy intake change mean±SD -85.5 kcal/day±465.4 in IG, and -132.0±513.2 in CG (*P*=0.41); - Triglycerides change mean±SD -9.9 mg/dL±87.7 in IG, and 8.6±144.8 in CG (*P*=0.22); - HbA1c change mean±SD 0.0%±0.1 in IG, and 0.1±0.2 in CG (*P*=0.15). |
| Palacios, C. [16] | 2018/USA | Baseline: 51 (27/24); 2-month follow-up: 32 (15/17) | IG: 3/24; CG: 2/22 | 33.8±7.30/36.8±5.86 | 35.6±7.50/33.3±5.81 | The MyNutriCart app was developed to guide individuals to make smart and healthy choices when purchasing foods at grocery stores, which provided a healthy grocery list based on the daily nutritional recommendations of the individuals that constitute the participant’s household. | This group received one face-to-face counseling session with an registered dietitian, and participants also received a sample menu. | 2 | - Weight changed from 93.3 kg to 92.9 in IG, and 83.3 to 82.8 in CG; - BMI changed from 35.6 kg/m^2^ to 35.4 in IG, and 33.3 to 33.1 in CG. |
| Patel, M. L. [17] | 2022/USA | Baseline: 38 (18/20); 1-month follow-up: 36 (17/19); 3-month follow-up: 35 (16/19) | IG: 2/16; CG: 4/16 | 44.6±12.5/49.9±15.2 | Total: 31.2±4.8 | Participants were instructed to self-monitor their dietary intake daily via the Fitbit mobile app, which allows users to track their calories of all foods and beverages consumed using a built-in nutritional database, barcode scanner, or manual entry of individual recipes and to graphically view their change in caloric intake. | Participants were instructed to self-monitor daily only highly caloric foods consumed that have limited nutritional value, referred to as red zone foods. | 3 | - Weight reduction -3.41 kg (95% CI -4.62 to -2.20) in IG, and -3.29 kg (95% CI -4.41 to -2.18) in CG. |
| Rogers, R. J. [18] | 2016/USA | Baseline: 39 (12/13/14); 3-month follow-up: 34 (12/9/13); 6-month follow-up: 27 (9/8/10) | IG1: 3/9; IG2: 3/10; CG: 2/12 | 40.6±9.5/39.5±9.7/39.7±10.4 | 39.7±2.9/39.3±3.2/39.5±2.6 | The BodyMedia® FIT System was utilized because the technology can potentially facilitate self-monitoring. Participants in the TECH group received a scheduled intervention telephone call one-time per month. | Standard behavioral weight loss attended weekly group meetings that were approximately 30–45 min in duration. | 6 | - Weight changed from 112.2 kg to 107.1 in IG1, 111.6 to 105.3 in IG2, and 110.9 to 104.4 in CG; - BMI changed from 39.7 kg/m^2^ to 37.8 in IG1, 39.3 to 37.3 in IG2, and 39.5 to 37.2 in CG; - WC changed from 122.8 cm to 118.2 in IG1, 122.3 to 115.3 in IG2, 119.1 to 115.1 in CG; - Fat mass changed from 51.3 kg to 47.7 in IG1, 50.6 to 45.4 in IG2, and 51.1 to 46.2 in CG. |
| Shin, D. W. [19] | 2017/Korea | Baseline: 35/35/35; 1-month follow-up: 35/33/32; 2-month follow-up: 34/32/32; 3-month follow-up: 34/32/32 | NA | 28.7±4.6/28.1±6.0/26.5±4.0 | 29.7±2.5/30.0±3.4/29.7±2.2 | A Fitmeter accelerometer and smart phone application customized for the intervention were provided to participants allocated to the two Smartcare arms. The application for IG2 included a feature designed to monitor and provide feedback of financial incentives. | Each participant received a one-to-one education on diet and exercise from a trained nurse for 5 minutes each session. | 3 | - Weight reduction mean ±SD -1.1 kg±2.9 in IG1, -3.1±3.7 in IG2, and -0.4±2.5 in CG; - BMI reduction mean±SD -0.5 kg/m^2^±0.8 in IG1, -1.0±1.2 in IG2, and -0.2±0.7 in CG; - WC reduction mean±SD -2.4 cm±2.3 in IG1, -4.4±3.3 in IG2, -1.2±3.0 in CG; - Fat mass reduction mean±SD -0.4 kg±2.1 in IG1, -2.8±3.3 in IG2, and -0.4±2.1 in CG; - SBP reduction mean±SD -4.7 mmHg±12.6 in IG1, -10.0±11.1 in IG2, and -2.3±11.1 in CG; - DBP reduction mean±SD -0.9 mmHg±7.0 in IG1, -4.2±9.1 in IG2, and 1.9±7.6 in CG; - Triglycerides change mean±SD 11.1±66.7 in IG1, -29.9±74.2 in IG2, and -14.8±165.5 in CG. |
| Simpson, S. A. [20] | 2020/UK | Baseline: 109 (73/36); 12-month follow-up: 84 (52/32) | IG: 24/49; CG: 9/27 | 46.2±10.6/49.4±10.7 | 37.8±6.0/37.1±5.7 | The website provided evidence-based information on weight loss, setting and monitoring goals, as well as advice on harnessing social support from family and/or friends. | The control group received a leaflet on healthy lifestyle and were offered access to the app and website after follow-up was complete. | 12 | - BMI change mean±SD -1.2 kg/m^2^±2.4 in IG, -0.9±3.3 in CG; - MVPA change mean±SD -8.5 min/day±32.2 in IG, -4.3±10.5 in CG. |
| Spring, B. [21] | 2017/USA | Baseline: 32/32/32; 3-month follow-up: 31/26/31; 6-month follow-up: 30/26/30; 12-month follow-up: 31/24/28 | IG1: 4/28; IG2: 6/26; CG: 5/27 | 40.4±10.7/37.3±13.3/40.1±11.1 | 34.8±2.8/34.8±3.0/34.3±3.2 | TECH participants were provided with study-designed ENGAGED app and accelerometer for 6 months. For the first 8 weeks, those attended weekly 90-minute group sessions and focused on nutrition, MVPA, and behavior change strategies. Weekly for the first 8 weeks and monthly from months 3–6, TECH and STND participants received calls which reviewed self-monitoring and goal attainment, and helped participants solve problems. | Those randomized to SELF attended one 60 minute group session at which treatment assignment was revealed and participants received their weight loss target, a calorie and fat gram counting book, and 6 months of daily paper self-monitoring diaries. They also received Group Lifestyle Balance DVDs. | 6 | - Weight reduction -4.7 (95% CI -6.7 to -2.5) in IG1, -6.6 (95% CI -8.8 to -4.4) in IG2, -2.7 (95% CI -5.1 to -0.3) in CG. |
| Thomas, J. G. [22] | 2017/USA | Baseline: 271 (94/91/86); 3-month follow-up: 83/81/76; 12-month follow-up: 84/75/75 | IG1: 22/72; IG2: 22/69; CG: 17/69 | 55.1±11.5/54.9±11.9/54.9±11.3 | 34.3±3.6/33.8±4.1/33.5±3.3 | Participants assigned to the WWO condition received access to WWO. Participants were instructed to access WWO for tracking daily food intake and PA, and weekly tracking of body weight. Participants assigned to the WWO plus ActiveLink (WWO1AL) condition received all of the resources in the WWO condition and an ActiveLink PA tracking device. | The Control condition consisted of online newsletters made available weekly for 3 months, then biweekly for 3 months, then monthly for 6 months. The newsletters contained general educational information on the benefits of losing weight and healthy eating and PA habits. | 12 | - Weight reduction -2.1 kg (95% CI -3.0 to -1.1) in IG1, -1.6 (95% CI -2.6 to -0.6) in IG2, and -1.2 (95% CI -2.3 to -0.2) in CG; - MVPA change -2.4 min/day (95% CI -10.0 to 5.2) in IG1, -1.3 (95% CI -8.9 to 6.3) in IG2, and 5.3 (95% CI -2.6 to 13.3) in CG. - Energy intake change -472.9 (95% CI -604.3 to -341.4) in IG1, -479.8 (95% -624.8 to -359.7) in IG2, -601.9 (-740.2 to -463.7) in CG. |
| Vaz, C. L. [23] | 2021/USA | Baseline: 13/15; 6-month follow-up: 13/15 | IG: 2/11; CG: 2/13 | 40.15±3.72/45.93±3.29 | 34.46±1.24/34.35±1.47 | The new electronically delivered lifestyle intervention is a smart phone app‐based monitoring and coaching system designed to enhance behavior modification with the goal of increasing energy expenditure and reducing energy intake. The intervention was designed to objectively track physical activity, weight, and diet. | No intervention. | 6 | - Weight difference -4.16 kg (95% CI -8.29 to -0.02) between IG and CG; - WC difference -5.70 cm (95% CI -9.91 to -1.50) between IG and CG; - SBP difference 4.36 mmHg (95% CI -9.06 to 17.79) between IG and CG; - DBP difference -3.23 mmHg (95% CI -10.18 to 3.71) between IG and CG; - HbA1c difference -0.25% mmHg (95% CI -0.44 to -0.06) between IG and CG; - Triglycerides difference 8.75 mg/dl (95% -30.91 to 48.41) between IG and CG. |
| Whitelock, V. [24] | 2019/UK | Baseline: 53/54; 1-month follow-up: 40/46; 2-month follow-up: 39/46 | IG: 12/41; CG: 16/38 | 42.8±10.5/44.5±10.7 | 35.9±6.8/35.2±6.2 | The attentive eating application was designed to promote attentive eating by encouraging users to photograph food and drink being consumed and then review this information when making dietary decisions throughout the day. Participants in the intervention group were also told they would receive an additional £10 compensation for continued use of the application. | Dietary advice booklet and weekly text messages only. | 2 | - Weight reduction mean±SD -1.2 kg±3.1 in IG, and -1.1 kg±3.4 in CG. |
| Allen, J. K. [25] | 2013/UK | Baseline: 18/16/17/17; 6-month follow-up: 12/11/10/10 | IG1: 4/14; IG2: 5/16; IG3: 4/13; IG4: 2/15 | 42.5±12.1/45.6±9.3/46.4±9.6/45.3±13.2 | 34.1±4.1/34.3±3.9/33.5±3.5/35.3±4.1 | Participants in the more intensive intervention groups received healthy eating and exercise counseling. The Lose It! weight loss application promoted self-management and mindful empowerment and provided real time feedback and motivators and opportunities for social networking and support. | NA | 6 | - Weight reduction mean±SD -2.5 kg±4.1 in IG1, -5.4±4.0 in IG2, -3.3±5.9 in IG3, -1.8±3.7 in IG4; - BMI reduction mean±SD -0.8 kg/m^2^±1.4 in IG1, -1.8±1.3 in IG2, -1.1±2.0 in IG3, -0.7±1.3 in IG4; - Male WC reduction mean±SD -3.0 cm±2.4 in IG1, -7.01±2.6 in IG2, -6.5±0.35 in IG3, -3.38±8.3 in IG4; - Female WC reduction mean±SD -3.19 cm±7.4 in IG1, -5.68±3.7 in IG2, -3.64±7.9 in IG3, -0.88±2.9 in IG4. |
| Ross, K. M. [26] | 2016/USA | Baseline: 80 (26/27/27); 6-month follow-up: 23/25/24 | IG1: 4/22; IG2: 3/24; IG3: 4/23 | 54.23±9.49/46.19±13.53/52.93±10.31 | NA | TECH condition, participants were given a Fitbit Zip activity monitor, a Fitbit Aria smart scale, and were asked to track their caloric intake using the Fitbit smartphone app. TECH+PHONE condition, participants additionally received phone-based interventionist contact. | Participants in the ST condition were provided with self-monitoring tools such as a calorie reference book, a pedometer and a body weight scale | 6 | - Weight reduction mean±SE -1.28 kg±1.19 in IG1, -4.04±1.37 in IG2, and -6.40±1.17 in CG. |
| Jospe, M. R. [27] | 2017/New Zealand | Baseline: 250 (50/51/50/51/48); 6-month follow-up: 36/48/40/36/44; 12-month follow-up: 28/39/36/32/36 | IG1: 19/32; IG2: 19/31; IG3: 20/31; IG4: 19/31; CG: 18/30 | 46.1±11.4/44.4±10.2/40.6±9.9/40.7±10.8/46.7±11.4 | 33.2±4.8/33.5±4.5/33±4.1/33±4.3/32.3±4.3 | Participants in the MyFitnessPal group were asked to track their diet. Participants were asked to track their dietary intake every day for the first month and for 1 week every month during months 2 to 12. | No intervention. | 12 | - Weight difference 0.8 kg (95% CI -2.1 to 3.7) between IG1 and CG, 2.2 (95% CI -1.0 to 5.3) between IG2 and CG, 1.7 (95% CI -0.9 to 4.3) between IG3 and CG, and -1.1 (95% CI -3.8 to 1.6) between IG4 and CG; - BMI difference 0.3 kg/m^2^ (95% CI -0.7 to 1.3) between IG1 and CG, 0.7 (95% CI -0.4 to 1.7) between IG2 and CG, 0.7 (95% CI -0.3 to 1.6) between IG3 and CG, and -0.3 (95% CI -1.3 to 0.6) between IG4 and CG; - WC difference 0.9 cm (95% CI -1.6 to 3.6) between IG1 and CG, 1.4 (95% CI -1.2 to 4.1) between IG2 and CG, 1.2 (95% CI -1.0 to 3.5) between IG3 and CG, and -0.3 (95% CI -2.9 to 2.3) between IG4 and CG; - Fat mass difference -0.7 kg (95% CI -3.3 to 1.8) between IG1 and CG, 0.3 (95% CI -2.3 to 2.9) between IG2 and CG, 0.7 (95% CI -1.9 to 3.4) between IG3 and CG, and -1.9 (95% CI -4.8 to 0.9) between IG4 and CG; - SBP difference 1.7 mmHg (95% CI -2.6 to 5.9) between IG1 and CG, 1.5 (95% CI -2.9 to 5.8) between IG2 and CG, -1.1 (95% CI -5.6 to 3.4) between IG3 and CG, and -0.5 (95% CI -5.1 to 4.1) between IG4 and CG; - DBP difference -1.4 mmHg (95% CI -4.6 to 1.8) between IG1 and CG, 1.9 (95% CI -1.4 to 5.2) between IG2 and CG, -1.4 (95% CI -4.8 to 2.1) between IG3 and CG, and -0.8 (95% CI -4.4 to 2.7) between IG4 and CG. |
| Jin, T. [28] | 2023/Korea | Baseline: 57 (30/27); 3-month follow-up: 29/25 | IG: 16/14; CG:14/13 | 25.0 ± 5.1/25.8 ± 4.7 | 28.2 ± 3.1/27.7 ± 2.2 | Participants were instructed to record their foods and supplements using the dietary self-monitoring tools. Participants were instructed to use a App for at least 20 days. | Participants were instructed to record their foods and supplements using the dietary self-monitoring tools. Participants were instructed to use a Paper for at least 20 days. | 3 | - Weight change from 81.4 kg to 82.1 in IG, and 78.5 to 77.9 in CG (*P*=0.11); - BMI change from 28.2 kg/m^2^ to 28.4 in IG, and 27.7 to 27.4 in CG (*P*=0.11); - WC change from 94.5 cm to 93.6 in IG, and 93.0 to 91.3 in CG (*P*=0.59); - Fat mass change from 26.2 kg to 27.3 in IG, and 26.5 to 26.0 in CG (*P*=0.03); - Triglycerides change from 92.1 mg/dL to 118.8 in IG, and 99.1 to 108.0 in CG (*P*=0.23). |
| Gemesi, K. [29] | 2024/Germany | Baseline: 168 (84/84); 3-month follow-up: 63/76 | IG: 27/57; CG: 33/51 | 47.4±11.5/46.3±10.6 | 34.3±2.5/34.1±3.0 | The DiHA “Oviva Direkt für Adipositas” delivers a 12-week multimodal weight loss intervention program according to the German guidelines for the prevention and treatment of obesity. | No intervention. | 3 | - Weight difference -2.3 kg (95% CI -3.1 to -1.4) between IG and CG; - Fat mass percentage difference -0.8% (95% CI -1.4 to -0.2) between IG and CG. |

Abbreviation: IG, intervention group; CG, control group; BMI, body mass index; HbA1c, hemoglobin A1c; PA, physical activity; MMM, my meal mate; EXP, experimental; CON, control; SBP, systolic blood pressure; DBP, diastolic blood pressure; WC, waist circumference; MVPA, moderate-to-vigorous physical activity; BPBH, Be Positive Be Healthe; CIMWM, companion-intensive multi-aspect weight management; TMWM, traditional multi-aspect weight management; TECH, technology; ENGAGED, e-networks guiding adherence to goals in exercise and diet; STND, standard; SELF, self-guided; WWO, weight watchers online; NA, not applicable.

References

1. Apiñaniz A, Cobos-Campos R, de Lafuente-Moríñigo AS, Parraza N, Aizpuru F, Pérez I, et al. Effectiveness of randomized controlled trial of a mobile app to promote healthy lifestyle in obese and overweight patients. Family Practice. 2019 Dec;36(6):699-705. PMID: WOS:000509468700005. doi: 10.1093/fampra/cmz020.

2. Bughin F, Bui G, Ayoub B, Blervaque L, Saey D, Avignon A, et al. Impact of a Mobile Telerehabilitation Solution on Metabolic Health Outcomes and Rehabilitation Adherence in Patients With Obesity: randomized Controlled Trial. JMIR mHealth and uHealth. 2021;9(12):e28242. PMID: CN-02358900. doi: 10.2196/28242.

3. Carter MC, Burley VJ, Nykjaer C, Cade JE. Adherence to a smartphone application for weight loss compared to website and paper diary: pilot randomized controlled trial. J Med Internet Res. 2013 Apr 15;15(4):e32. PMID: 23587561. doi: 10.2196/jmir.2283.

4. Choi JH, Kim SW, Seo J, Sun Y, Jung WS, Park HY, et al. Effects of a Mobile-Health Exercise Intervention on Body Composition, Vascular Function, and Autonomic Nervous System Function in Obese Women: A Randomized Controlled Trial. J Multidiscip Healthc. 2023;16:1601-15. PMID: 37313274. doi: 10.2147/jmdh.S406905.

5. Domal SV, Chandrasekaran B, Palanisamy HP. Influence of smartphone-based physical activity intervention on executive functions and cardiometabolic disease risk in obese young adults: a pilot randomised controlled trial. J Diabetes Metab Disord. 2023 Jun;22(1):619-28. PMID: 37255799. doi: 10.1007/s40200-023-01182-9.

6. Duncan MJ, Fenton S, Brown WJ, Collins CE, Glozier N, Kolt GS, et al. Efficacy of a Multi-component m-Health Weight-loss Intervention in Overweight and Obese Adults: A Randomised Controlled Trial. Int J Environ Res Public Health. 2020 Aug 26;17(17). PMID: 32859100. doi: 10.3390/ijerph17176200.

7. Godino JG, Merchant G, Norman GJ, Donohue MC, Marshall SJ, Fowler JH, et al. Using social and mobile tools for weight loss in overweight and obese young adults (Project SMART): a 2 year, parallel-group, randomised, controlled trial. Lancet Diabetes Endocrinol. 2016 Sep;4(9):747-55. PMID: 27426247. doi: 10.1016/s2213-8587(16)30105-x.

8. Hebden L, Cook A, van der Ploeg HP, King L, Bauman A, Allman-Farinelli M. A mobile health intervention for weight management among young adults: a pilot randomised controlled trial. J Hum Nutr Diet. 2014 Aug;27(4):322-32. PMID: 23992038. doi: 10.1111/jhn.12155.

9. Hurkmans E, Matthys C, Bogaerts A, Scheys L, Devloo K, Seghers J. Face-to-Face Versus Mobile Versus Blended Weight Loss Program: Randomized Clinical Trial. JMIR Mhealth Uhealth. 2018 Jan 11;6(1):e14. PMID: 29326093. doi: 10.2196/mhealth.7713.

10. Hutchesson MJ, Callister R, Morgan PJ, Pranata I, Clarke ED, Skinner G, et al. A Targeted and Tailored eHealth Weight Loss Program for Young Women: The Be Positive Be Healthe Randomized Controlled Trial. Healthcare (Basel). 2018 May 2;6(2). PMID: 29724054. doi: 10.3390/healthcare6020039.

11. Jiang WZ, Huang SS, Ma S, Gong YY, Fu ZZ, Zhou L, et al. Effectiveness of companion-intensive multi-aspect weight management in Chinese adults with obesity: a 6-month multicenter randomized clinical trial. Nutrition & Metabolism. 2021 Jan;18(1). PMID: WOS:000614419300001. doi: 10.1186/s12986-020-00511-6.

12. Kliemann N, Croker H, Johnson F, Beeken RJ. Development of the Top Tips Habit-Based Weight Loss App and Preliminary Indications of Its Usage, Effectiveness, and Acceptability: Mixed-Methods Pilot Study. Jmir Mhealth and Uhealth. 2019 May;7(5). PMID: WOS:000467847700001. doi: 10.2196/12326.

13. Lugones-Sanchez C, Recio-Rodriguez JI, Agudo-Conde C, Repiso-Gento I, E GA, Ramirez-Manent JI, et al. Long-term Effectiveness of a Smartphone App Combined With a Smart Band on Weight Loss, Physical Activity, and Caloric Intake in a Population With Overweight and Obesity (Evident 3 Study): Randomized Controlled Trial. J Med Internet Res. 2022 Feb 1;24(2):e30416. PMID: 35103609. doi: 10.2196/30416.

14. Lugones-Sanchez C, Sanchez-Calavera MA, Repiso-Gento I, Adalia EG, Ramirez-Manent JI, Agudo-Conde C, et al. Effectiveness of an mHealth Intervention Combining a Smartphone App and Smart Band on Body Composition in an Overweight and Obese Population: Randomized Controlled Trial (EVIDENT 3 Study). JMIR Mhealth Uhealth. 2020 Nov 26;8(11):e21771. PMID: 33242020. doi: 10.2196/21771.

15. Nakata Y, Sasai H, Gosho M, Kobayashi H, Shi Y, Ohigashi T, et al. A Smartphone Healthcare Application, CALO mama Plus, to Promote Weight Loss: A Randomized Controlled Trial. Nutrients. 2022 Nov 2;14(21). PMID: 36364870. doi: 10.3390/nu14214608.

16. Palacios C, Torres M, López D, Trak-Fellermeier MA, Coccia C, Pérez CM. Effectiveness of the Nutritional App "MyNutriCart" on Food Choices Related to Purchase and Dietary Behavior: A Pilot Randomized Controlled Trial. Nutrients. 2018 Dec 12;10(12). PMID: 30545125. doi: 10.3390/nu10121967.

17. Patel ML, Cleare AE, Smith CM, Rosas LG, King AC. Detailed Versus Simplified Dietary Self-monitoring in a Digital Weight Loss Intervention Among Racial and Ethnic Minority Adults: Fully Remote, Randomized Pilot Study. JMIR Form Res. 2022 Dec 13;6(12):e42191. PMID: 36512404. doi: 10.2196/42191.

18. Rogers RJ, Lang W, Barone Gibbs B, Davis KK, Burke LE, Kovacs SJ, et al. Applying a technology-based system for weight loss in adults with obesity. Obesity science and practice. 2016;2(1):3‐12. PMID: CN-01786336. doi: 10.1002/osp4.18.

19. Shin DW, Yun JM, Shin JH, Kwon H, Min HY, Joh HK, et al. Enhancing physical activity and reducing obesity through smartcare and financial incentives: A pilot randomized trial. Obesity (Silver Spring). 2017 Feb;25(2):302-10. PMID: 28063226. doi: 10.1002/oby.21731.

20. Simpson SA, Matthews L, Pugmire J, McConnachie A, McIntosh E, Coulman E, et al. An app-, web- and social support-based weight loss intervention for adults with obesity: the ‘HelpMeDoIt!’ feasibility randomised controlled trial. Pilot and feasibility studies. 2020;6(1). PMID: CN-02422283. doi: 10.1186/s40814-020-00656-4.

21. Spring B, Pellegrini CA, Pfammatter A, Duncan JM, Pictor A, McFadden HG, et al. Effects of an abbreviated obesity intervention supported by mobile technology: The ENGAGED randomized clinical trial. Obesity (Silver Spring). 2017 Jul;25(7):1191-8. PMID: 28494136. doi: 10.1002/oby.21842.

22. Thomas JG, Raynor HA, Bond DS, Luke AK, Cardoso CC, Foster GD, et al. Weight loss in Weight Watchers Online with and without an activity tracking device compared to control: A randomized trial. Obesity (Silver Spring). 2017 Jun;25(6):1014-21. PMID: 28437597. doi: 10.1002/oby.21846.

23. Vaz CL, Carnes N, Pousti B, Zhao H, Williams KJ. A randomized controlled trial of an innovative, user-friendly, interactive smartphone app-based lifestyle intervention for weight loss. Obesity Science and Practice. 2021;7(5):555-68. doi: 10.1002/osp4.503.

24. Whitelock V, Kersbergen I, Higgs S, Aveyard P, Halford JCG, Robinson E. A smartphone based attentive eating intervention for energy intake and weight loss: results from a randomised controlled trial. BMC Public Health. 2019 May 21;19(1):611. PMID: 31113400. doi: 10.1186/s12889-019-6923-x.

25. Allen JK, Stephens J, Dennison Himmelfarb CR, Stewart KJ, Hauck S. Randomized controlled pilot study testing use of smartphone technology for obesity treatment. J Obes. 2013;2013:151597. PMID: 24392223. doi: 10.1155/2013/151597.

26. Ross KM, Wing RR. Impact of newer self-monitoring technology and brief phone-based intervention on weight loss: A randomized pilot study. Obesity (Silver Spring). 2016 Aug;24(8):1653-9. PMID: 27367614. doi: 10.1002/oby.21536.

27. Jospe MR, Roy M, Brown RC, Williams SM, Osborne HR, Meredith-Jones KA, et al. The Effect of Different Types of Monitoring Strategies on Weight Loss: A Randomized Controlled Trial. Obesity. 2017 Sep;25(9):1490-8. PMID: WOS:000408479300007. doi: 10.1002/oby.21898.

28. Jin T, Kang G, Song S, Lee H, Chen Y, Kim SE, et al. The effects of dietary self-monitoring intervention on anthropometric and metabolic changes via a mobile application or paper-based diary: a randomized trial. Nutr Res Pract. 2023 Dec;17(6):1238-54. PMID: 38053827. doi: 10.4162/nrp.2023.17.6.1238.

29. Gemesi K, Winkler S, Schmidt-Tesch S, Schederecker F, Hauner H, Holzapfel C. Efficacy of an app-based multimodal lifestyle intervention on body weight in persons with obesity: results from a randomized controlled trial. Int J Obes (Lond). 2024 Jan;48(1):118-26. PMID: 38017117. doi: 10.1038/s41366-023-01415-0.
