# Supplementary material for: Behavior Change Resources Used in Mobile App–Based Interventions Addressing Weight, Behavioral, and Metabolic Outcomes in Adults With Overweight and Obesity: Systematic Review and Meta-Analysis of Randomized Controlled Trials
Source: JMIR Mhealth Uhealth. 2025 Aug 19;13:e63313. doi: 10.2196/63313 (PMC12392691; doi:10.2196/63313)
Supplement: Multimedia Appendix 5 [file mhealth-v13-e63313-s005.docx]

| Table S5.1 BCTs identified in the intervention group of each study | | | | | | | | | | |
| --- | --- | --- | --- | --- | --- | --- | --- | --- | --- | --- |
| **BCT taxonomy** | Apiñaniz, A., 2019 | Bughin, F.,  2021 | Carter, M. C., 2013 | Choi, J. H., 2023 | Domal, S. V., 2023 | Duncan, M. J., 2020 | Godino, J. G., 2016 | Hebden, L., 2014 | Hurkmans, E., 2018 | Hutchesson, M. J., 2018 |
| 1.1 Goal setting (behaviour) | **√** | **√** |  | **√** | **√** | **√** | **√** |  |  | **√** |
| 1.2 Problem solving |  |  |  |  |  | **√** |  |  |  | **√** |
| 1.3 Goal setting (outcome) |  |  | **√** |  |  |  |  |  |  | **√** |
| 1.4 Action planning | **√** | **√** |  | **√** | **√** | **√** |  |  |  | **√** |
| 1.5 Review behaviour goal(s) |  |  | **√** |  |  |  | **√** |  |  |  |
| 1.7 Review outcome goal(s) |  |  |  |  |  |  |  |  |  | **√** |
| 2.1 Monitoring of behaviour by others without feedback |  | **√** | **√** |  |  |  |  |  |  |  |
| 2.2 Feedback on behaviour |  | **√** | **√** | **√** | **√** | **√** | **√** | **√** |  | **√** |
| 2.3 Self-monitoring of behaviour | **√** | **√** | **√** |  |  | **√** | **√** | **√** | **√** | **√** |
| 2.4 Self-monitoring of outcome(s) of behaviour |  |  |  |  |  | **√** |  |  |  | **√** |
| 2.5 Monitoring of outcome(s) of behaviour without feedback |  |  |  |  |  |  |  |  |  | **√** |
| 2.6 Biofeedback |  | **√** |  | **√** |  | **√** |  |  |  |  |
| 2.7 Feedback on outcome(s) of behaviour |  |  |  |  |  | **√** | **√** |  |  |  |
| 3.1 Social support (unspecified) | **√** |  |  |  |  | **√** | **√** | **√** | **√** | **√** |
| 3.2 Social support (practical) |  | **√** |  |  |  |  |  |  |  |  |
| 3.3 Social support (emotional) |  |  | **√** |  |  |  |  |  |  |  |
| 4.1 Instruction on how to perform the behaviour | **√** | **√** |  |  |  | **√** | **√** | **√** | **√** | **√** |
| 5.1 Information about health consequences | **√** | **√** |  |  |  | **√** | **√** |  |  |  |
| 6.1 Demonstration of the behaviour | **√** | **√** |  | **√** |  |  |  |  |  |  |
| 6.2 Social comparison |  |  |  |  |  |  |  |  |  |  |
| 7.1 Prompts/cues |  | **√** |  |  |  | **√** | **√** | **√** |  | **√** |
| 8.1 Behavioural practice/rehearsal |  |  |  |  |  |  | **√** |  |  |  |
| 8.3 Habit formation |  |  |  |  |  |  | **√** |  |  |  |
| 8.7 Graded tasks |  |  |  |  | **√** |  |  |  |  |  |
| 10.1 Material incentive (behavior) |  |  |  |  |  |  |  |  |  |  |
| 10.3 Non-specific reward |  |  |  |  |  |  |  |  |  | **√** |
| 10.4 Social reward |  |  |  |  |  |  |  | **√** |  |  |
| 10.8 Incentive (outcome) |  |  |  |  |  |  |  |  |  |  |
| 10.9 Self-reward |  |  |  |  |  |  |  |  |  | **√** |
| 11.2 Reduce negative emotions |  |  |  |  |  | **√** |  |  |  |  |
| 11.3 Conserving mental resources |  |  | **√** |  |  |  | **√** |  |  |  |
| 12.5 Adding objects to the environment |  | **√** |  | **√** |  | **√** |  |  |  |  |
| 14.4 Reward approximation |  |  |  |  |  |  |  |  |  |  |
| 15.4 Self-talk |  |  | **√** |  |  |  |  |  |  |  |
| **Total No. of BCT** | **7** | **12** | **8** | **6** | **4** | **14** | **12** | **6** | **3** | **14** |

| Table S5.1 BCTs identified in the intervention group of each study (continued) | | | | | | | | | | |
| --- | --- | --- | --- | --- | --- | --- | --- | --- | --- | --- |
| **BCT taxonomy** | Jiang, W. Z., 2021 | Kliemann, N., 2019 | Lugones-Sanchez, C., 2022 | Lugones-Sanchez, C., 2020 | Nakata, Y., 2022 | Palacios, C., 2018 | Patel, M. L., 2022 | Rogers, R. J., 2016 | Shin, D. W., 2017 | Simpson, S. A., 2017 |
| 1.1 Goal setting (behaviour) |  |  | **√** | **√** | **√** |  | **√** |  |  |  |
| 1.2 Problem solving |  |  |  |  |  |  | **√** |  |  | **√** |
| 1.3 Goal setting (outcome) |  |  |  |  |  |  | **√** |  |  | **√** |
| 1.4 Action planning | **√** | **√** | **√** | **√** |  |  | **√** |  | **√** | **√** |
| 1.5 Review behaviour goal(s) |  | **√** |  |  | **√** |  |  |  |  |  |
| 1.7 Review outcome goal(s) |  |  |  |  |  |  |  |  |  | **√** |
| 2.1 Monitoring of behaviour by others without feedback |  |  |  |  |  |  |  |  |  |  |
| 2.2 Feedback on behaviour | **√** |  | **√** | **√** | **√** |  | **√** | **√** |  |  |
| 2.3 Self-monitoring of behaviour | **√** | **√** | **√** | **√** | **√** |  | **√** | **√** | **√** |  |
| 2.4 Self-monitoring of outcome(s) of behaviour | **√** | **√** |  |  | **√** |  | **√** | **√** |  | **√** |
| 2.5 Monitoring of outcome(s) of behaviour without feedback |  |  |  |  |  |  |  |  |  |  |
| 2.6 Biofeedback |  |  |  |  |  |  |  |  |  |  |
| 2.7 Feedback on outcome(s) of behaviour | **√** |  |  |  | **√** |  | **√** | **√** |  | **√** |
| 3.1 Social support (unspecified) | **√** |  | **√** | **√** |  |  | **√** |  |  | **√** |
| 3.2 Social support (practical) |  |  |  |  |  |  |  | **√** | **√** | **√** |
| 3.3 Social support (emotional) |  |  |  |  |  |  |  |  |  | **√** |
| 4.1 Instruction on how to perform the behaviour | **√** | **√** | **√** | **√** | **√** | **√** | **√** | **√** | **√** | **√** |
| 5.1 Information about health consequences |  |  | **√** | **√** |  |  |  |  | **√** |  |
| 6.1 Demonstration of the behaviour |  |  |  |  |  |  |  |  |  |  |
| 6.2 Social comparison |  |  |  |  |  |  |  |  |  | **√** |
| 7.1 Prompts/cues |  | **√** |  |  | **√** |  | **√** |  |  | **√** |
| 8.1 Behavioural practice/rehearsal |  |  |  |  |  |  |  |  |  |  |
| 8.3 Habit formation |  | **√** |  |  |  |  |  |  |  |  |
| 8.7 Graded tasks |  |  |  |  |  |  | **√** |  |  |  |
| 10.1 Material incentive (behavior) |  |  |  |  |  |  |  |  | **√** |  |
| 10.3 Non-specific reward |  |  |  |  |  |  |  |  |  | **√** |
| 10.4 Social reward |  |  | **√** | **√** | **√** |  |  |  |  |  |
| 10.8 Incentive (outcome) |  |  |  |  |  |  |  |  | **√** |  |
| 10.9 Self-reward |  |  |  |  |  |  |  |  |  |  |
| 11.2 Reduce negative emotions |  |  |  |  |  |  |  |  |  |  |
| 11.3 Conserving mental resources |  |  |  |  |  | **√** |  |  |  |  |
| 12.5 Adding objects to the environment |  |  | **√** | **√** |  |  | **√** | **√** | **√** |  |
| 14.4 Reward approximation |  |  |  |  |  |  |  |  |  |  |
| 15.4 Self-talk |  |  |  |  |  |  |  |  |  |  |
| **Total No. of BCT** | **7** | **7** | **9** | **9** | **9** | **2** | **13** | **7** | **8** | **13** |

| Table S5.1 BCTs identified in the intervention group of each study (continued) | | | | | | | | | | |
| --- | --- | --- | --- | --- | --- | --- | --- | --- | --- | --- |
| **BCT taxonomy** | Spring, B., 2017 | Thomas, J. G., 2017 | Vaz, C. L., 2021 | Whitelock, V., 2019 | Allen, J. K., 2013 | Ross, K. M., 2016 | Jospe, M. R., 2017 | Jin, T., 2023 | Gemesi, K., 2024 |  |
| 1.1 Goal setting (behaviour) | **√** | **√** | **√** |  | **√** | **√** | **√** | **√** | **√** |  |
| 1.2 Problem solving | **√** |  | **√** |  |  | **√** |  |  |  |  |
| 1.3 Goal setting (outcome) | **√** |  | **√** |  | **√** |  |  |  | **√** |  |
| 1.4 Action planning |  |  | **√** |  |  |  | **√** |  |  |  |
| 1.5 Review behaviour goal(s) | **√** |  |  |  |  |  |  |  |  |  |
| 1.7 Review outcome goal(s) | **√** |  | **√** |  |  |  |  |  |  |  |
| 2.1 Monitoring of behaviour by others without feedback |  |  |  |  |  |  |  |  |  |  |
| 2.2 Feedback on behaviour | **√** |  | **√** | **√** | **√** | **√** |  |  | **√** |  |
| 2.3 Self-monitoring of behaviour | **√** | **√** | **√** | **√** | **√** | **√** | **√** | **√** | **√** |  |
| 2.4 Self-monitoring of outcome(s) of behaviour | **√** | **√** | **√** |  | **√** | **√** |  |  | **√** |  |
| 2.5 Monitoring of outcome(s) of behaviour without feedback |  |  |  |  |  |  |  |  |  |  |
| 2.6 Biofeedback |  |  |  |  |  |  |  |  |  |  |
| 2.7 Feedback on outcome(s) of behaviour | **√** | **√** | **√** |  | **√** | **√** |  |  | **√** |  |
| 3.1 Social support (unspecified) | **√** |  | **√** |  | **√** | **√** |  |  | **√** |  |
| 3.2 Social support (practical) | **√** |  |  |  | **√** |  |  |  |  |  |
| 3.3 Social support (emotional) |  |  | **√** |  |  |  |  | **√** | **√** |  |
| 4.1 Instruction on how to perform the behaviour | **√** |  | **√** | **√** | **√** |  | **√** | **√** | **√** |  |
| 5.1 Information about health consequences |  |  |  |  |  |  |  |  |  |  |
| 6.1 Demonstration of the behaviour |  |  |  |  |  |  |  |  |  |  |
| 6.2 Social comparison | **√** |  | **√** |  |  |  |  |  |  |  |
| 7.1 Prompts/cues | **√** |  | **√** | **√** |  |  |  |  | **√** |  |
| 8.1 Behavioural practice/rehearsal |  |  |  | **√** |  |  |  |  |  |  |
| 8.3 Habit formation |  |  |  |  |  |  |  |  |  |  |
| 8.7 Graded tasks |  |  |  |  |  |  |  |  |  |  |
| 10.1 Material incentive (behavior) | **√** |  |  | **√** |  |  |  |  |  |  |
| 10.3 Non-specific reward |  |  | **√** | **√** |  |  |  |  |  |  |
| 10.4 Social reward |  | **√** |  |  |  |  |  |  |  |  |
| 10.8 Incentive (outcome) |  |  |  |  |  |  |  |  |  |  |
| 10.9 Self-reward |  |  |  |  |  |  |  |  |  |  |
| 11.2 Reduce negative emotions |  |  |  |  |  |  |  |  |  |  |
| 11.3 Conserving mental resources |  |  |  |  | **√** |  |  |  |  |  |
| 12.5 Adding objects to the environment | **√** | **√** | **√** |  |  | **√** |  |  |  |  |
| 14.4 Reward approximation |  | **√** |  |  |  |  |  |  |  |  |
| 15.4 Self-talk |  |  |  |  |  |  |  |  |  |  |
| **Total No. of BCT** | **16** | **7** | **16** | **7** | **10** | **8** | **4** | **4** | **10** |  |

Abbreviation: BCT, behavior change technique.

| Table S5.2 BCTs identified in the control group of each study | | | | | | | | | | |
| --- | --- | --- | --- | --- | --- | --- | --- | --- | --- | --- |
| **BCT taxonomy** | Apiñaniz, A., 2019 | Bughin, F.,  2021 | Carter, M. C., 2013 | Choi, J. H., 2023 | Domal, S. V., 2023 | Duncan, M. J., 2020 | Godino, J. G., 2016 | Hebden, L., 2014 | Hurkmans, E., 2018 | Hutchesson, M. J., 2018 |
| 1.1 Goal setting (behaviour) | **√** |  | **√** |  |  |  |  |  |  |  |
| 1.2 Problem solving |  |  |  |  |  |  |  |  |  |  |
| 1.3 Goal setting (outcome) |  |  |  |  |  |  |  |  |  |  |
| 1.4 Action planning | **√** |  |  |  |  |  |  |  |  |  |
| 1.5 Review behaviour goal(s) |  |  |  |  |  |  |  |  |  |  |
| 1.7 Review outcome goal(s) |  |  |  |  |  |  |  |  |  |  |
| 2.2 Feedback on behaviour |  |  |  |  |  |  |  |  |  |  |
| 2.3 Self-monitoring of behaviour |  |  | **√** |  |  |  |  |  |  |  |
| 2.4 Self-monitoring of outcome(s) of behaviour |  |  |  |  |  |  |  |  |  |  |
| 2.7 Feedback on outcome(s) of behaviour |  |  |  |  |  |  |  |  |  |  |
| 3.1 Social support (unspecified) |  |  |  |  |  |  |  |  |  |  |
| 3.2 Social support (practical) |  | **√** |  |  |  |  |  |  |  |  |
| 3.3 Social support (emotional) |  |  |  |  |  |  |  |  |  |  |
| 4.1 Instruction on how to perform the behaviour | **√** | **√** |  |  |  |  |  | **√** |  |  |
| 5.1 Information about health consequences |  |  |  |  |  |  | **√** |  |  |  |
| 6.1 Demonstration of the behaviour |  |  |  |  |  |  |  |  |  |  |
| 7.1 Prompts/cues |  |  |  |  |  |  |  |  |  |  |
| 11.3 Conserving mental resources |  |  |  |  |  |  |  |  |  |  |
| 12.5 Adding objects to the environment |  |  |  |  |  |  |  |  |  |  |
| **Total No. of BCT** | **3** | **3** | **2** | **0** | **0** | **0** | **1** | **1** | **0** | **0** |

| Table S5.2 BCTs identified in the control group of each study (continued) | | | | | | | | | | |
| --- | --- | --- | --- | --- | --- | --- | --- | --- | --- | --- |
| **BCT taxonomy** | Jiang, W. Z., 2021 | Kliemann, N., 2019 | Lugones-Sanchez, C., 2022 | Lugones-Sanchez, C., 2020 | Nakata, Y., 2022 | Palacios, C., 2018 | Patel, M. L., 2022 | Rogers, R. J., 2016 | Shin, D. W., 2017 | Simpson, S. A., 2017 |
| 1.1 Goal setting (behaviour) |  |  |  |  |  |  | **√** | **√** |  |  |
| 1.2 Problem solving |  |  |  |  |  |  | **√** | **√** |  |  |
| 1.3 Goal setting (outcome) |  |  |  |  |  |  | **√** |  |  |  |
| 1.4 Action planning | **√** |  | **√** | **√** |  |  | **√** |  |  |  |
| 1.5 Review behaviour goal(s) |  |  |  |  |  |  |  | **√** |  |  |
| 1.7 Review outcome goal(s) |  |  |  |  |  |  | **√** |  |  |  |
| 2.2 Feedback on behaviour |  |  |  |  |  |  | **√** | **√** |  |  |
| 2.3 Self-monitoring of behaviour | **√** |  |  |  |  |  | **√** | **√** |  |  |
| 2.4 Self-monitoring of outcome(s) of behaviour | **√** |  |  |  |  |  | **√** |  |  |  |
| 2.7 Feedback on outcome(s) of behaviour |  |  |  |  |  |  | **√** | **√** |  |  |
| 3.1 Social support (unspecified) | **√** |  | **√** | **√** |  |  | **√** |  |  |  |
| 3.2 Social support (practical) |  |  |  |  |  | **√** |  |  |  |  |
| 3.3 Social support (emotional) |  |  |  |  |  |  |  |  |  |  |
| 4.1 Instruction on how to perform the behaviour | **√** |  | **√** | **√** |  | **√** | **√** | **√** | **√** | **√** |
| 5.1 Information about health consequences |  |  | **√** | **√** |  |  |  |  |  |  |
| 6.1 Demonstration of the behaviour |  |  |  |  |  |  |  |  |  |  |
| 7.1 Prompts/cues |  |  |  |  |  |  | **√** |  |  |  |
| 11.3 Conserving mental resources |  |  |  |  |  |  | **√** |  |  |  |
| 12.5 Adding objects to the environment |  |  |  |  |  |  | **√** |  |  |  |
| **Total No. of BCT** | **5** | **0** | **4** | **4** | **0** | **2** | **14** | **7** | **1** | **1** |

| Table S5.2 BCTs identified in the control group of each study (continued) | | | | | | | | | | |
| --- | --- | --- | --- | --- | --- | --- | --- | --- | --- | --- |
| **BCT taxonomy** | Spring, B., 2017 | Thomas, J. G., 2017 | Vaz, C. L., 2021 | Whitelock, V., 2019 | Allen, J. K., 2013 | Ross, K. M., 2016 | Jospe, M. R., 2017 | Jin, T., 2023 | Gemesi, K., 2024 |  |
| 1.1 Goal setting (behaviour) | **√** |  |  |  | **√** |  |  | **√** |  |  |
| 1.2 Problem solving |  |  |  |  |  |  |  |  |  |  |
| 1.3 Goal setting (outcome) | **√** |  |  |  | **√** |  |  |  |  |  |
| 1.4 Action planning |  |  |  |  |  |  | **√** |  |  |  |
| 1.5 Review behaviour goal(s) |  |  |  |  |  |  |  |  |  |  |
| 1.7 Review outcome goal(s) |  |  |  |  |  |  |  |  |  |  |
| 2.2 Feedback on behaviour |  |  |  |  |  |  |  |  |  |  |
| 2.3 Self-monitoring of behaviour | **√** |  |  |  |  | **√** |  | **√** |  |  |
| 2.4 Self-monitoring of outcome(s) of behaviour | **√** |  |  |  |  | **√** |  |  |  |  |
| 2.7 Feedback on outcome(s) of behaviour |  |  |  |  |  |  |  |  |  |  |
| 3.1 Social support (unspecified) |  |  |  |  |  |  |  |  |  |  |
| 3.2 Social support (practical) |  |  |  |  | **√** |  |  |  |  |  |
| 3.3 Social support (emotional) |  |  |  |  |  |  |  | **√** |  |  |
| 4.1 Instruction on how to perfor10  m the behaviour | **√** | **√** |  | **√** | **√** | **√** | **√** | **√** |  |  |
| 5.1 Information about health consequences |  | **√** |  | **√** |  |  |  |  |  |  |
| 6.1 Demonstration of the behaviour | **√** |  |  |  |  |  |  |  |  |  |
| 7.1 Prompts/cues |  |  |  | **√** |  |  |  |  |  |  |
| 11.3 Conserving mental resources |  |  |  |  |  |  |  |  |  |  |
| 12.5 Adding objects to the environment |  |  |  |  |  | **√** |  |  |  |  |
| **Total No. of BCT** | **6** | **2** | **0** | **3** | **4** | **4** | **2** | **4** | **0** |  |

Abbreviation: BCT, behavior change technique.
