# Supplementary material for: Behavior Change Resources Used in Mobile App–Based Interventions Addressing Weight, Behavioral, and Metabolic Outcomes in Adults With Overweight and Obesity: Systematic Review and Meta-Analysis of Randomized Controlled Trials
Source: JMIR Mhealth Uhealth. 2025 Aug 19;13:e63313. doi: 10.2196/63313 (PMC12392691; doi:10.2196/63313)
Supplement: Multimedia Appendix 6 [file mhealth-v13-e63313-s006.docx]

| Table S6 Mapping of Behavior Change Techniques (BCTs) to Resource Types | | |
| --- | --- | --- |
| **BCTs** | **Resource types** | **Explanation** |
| 1. Goals and planning | boosting | All BCTs under the "Goals and Planning" category are classified as boosting because they primarily involve cognitive engagement and the enhancement of reflective internal resources. |
| 1.1 Goal setting (behaviour) |  |  |
| 1.2 Problem solving |  |  |
| 1.3 Goal setting (outcome) |  |  |
| 1.4 Action planning |  |  |
| 1.5 Review behaviour goal(s) |  |  |
| 1.7 Review outcome goal(s) |  |  |
| 2. Feedback and monitoring | | |
| 2.1 Monitoring of behaviour by others without feedback | nudging | This BCT involves the observation or recording of an individual’s behaviour by others without providing feedback. The primary mechanism is the subtle influence on the individual's behaviour through the awareness of being monitored, which changes the affective resource. |
| 2.2 Feedback on behaviour | facilitating | Feedback on behaviour involves providing individuals with information about their performance from an external source. |
| 2.3 Self-monitoring of behaviour | boosting | Self-monitoring of behaviour requires individuals to observe and record their own behaviours, engaging their reflective internal resources. |
| 2.4 Self-monitoring of outcome(s) of behaviour | boosting | Similar to self-monitoring of behaviour. |
| 2.5 Monitoring of outcome(s) of behaviour without feedback | nudging | Similar to monitoring of behaviour by others without feedback. |
| 2.6 Biofeedback | facilitating | Biofeedback involves providing individuals with physiological information about their bodies using external monitoring devices. |
| 2.7 Feedback on outcome(s) of behaviour | facilitating | Similar to feedback on behaviour. |
| 3. Social support | facilitating | All BCTs under the "Social Support" category are classified as Facilitating because they primarily rely on external resources provided by others. |
| 3.1 Social support (unspecified) |  |  |
| 3.2 Social support (practical) |  |  |
| 3.3 Social support (emotional) |  |  |
| 4. Shaping knowledge | | |
| 4.1 Instruction on how to perform the behaviour | boosting | This BCT involves providing detailed guidance on how to execute a specific behavior, typically through skill training. While it requires external resources such as instructors or training materials, its primary focus is on enhancing the individual's internal reflective resources, including knowledge, skills. |
| 5. Natural consequences | | |
| 5.1 Information about health consequences | boosting | This BCT involves providing individuals with information about the health consequences of certain behaviors. The primary mechanism is to enhance the individual's knowledge and awareness about the impacts of their behavior, which is a reflective internal resource. |
| 6. Comparison of behaviour | | |
| 6.1 Demonstration of the behaviour | boosting | Similar to instruction on how to perform the behavior. |
| 6.2 Social comparison | nudging | The primary mechanism is the influence of social norms and peer behavior, which affects the individual's affective resources such as motivation and emotions. By leveraging the social context and the desire to conform or compete with others, individuals are subtly nudged towards behavior change. |
| 7. Associations | | |
| 7.1 Prompts/cues | nudging | This BCT involves introducing environmental or social stimuli that serve as triggers for the desired behavior. The primary mechanism is to subtly influence behavior by activating affective components of decision-making. |
| 8. Repetition and substitution | | |
| 8.1 Behavioural practice/rehearsal | boosting | All BCTs under the "Repetition and substitution" category are classified as Boosting because they primarily involve cognitive engagement and the enhancement of reflective internal resources. |
| 8.3 Habit formation |  |  |
| 8.7 Graded tasks |  |  |
| 10. Reward and threat | | |
| 10.1 Material incentive (behavior) | nudging | All BCTs under the "Reward and threat" category are classified as Nudging because they primarily use internal or external resources to create positive emotional and motivational responses. |
| 10.3 Non-specific reward |  |  |
| 10.4 Social reward |  |  |
| 10.8 Incentive (outcome) |  |  |
| 10.9 Self-reward |  |  |
| 11. Regulation | | |
| 11.2 Reduce negative emotions | nudging | Both BCTs under the "Regulation" category are classified as Nudging because they primarily aim to influence behavior by leveraging affective resources. |
| 11.3 Conserving mental resources |  |  |
| 12. Antecedents | | |
| 12.5 Adding objects to the environment | facilitating | The primary mechanism is the provision of external resources that make the desired behavior more accessible or likely. |
| 14. Scheduled consequences | | |
| 14.4 Reward approximation | nudging | The primary mechanism is to influence behavior through positive reinforcement, creating a positive emotional response that encourages further progress. |
| 15. Self-belief | | |
| 15.4 Self-talk | nudging | The primary mechanism is to influence behavior through emotional and motivational self-regulation, leveraging internal affective resources. |
