# Supplementary figures and images for: Behavior Change Resources Used in Mobile App–Based Interventions Addressing Weight, Behavioral, and Metabolic Outcomes in Adults With Overweight and Obesity: Systematic Review and Meta-Analysis of Randomized Controlled Trials
Source: JMIR Mhealth Uhealth. 2025 Aug 19;13:e63313. doi: 10.2196/63313 (PMC12392691; doi:10.2196/63313)

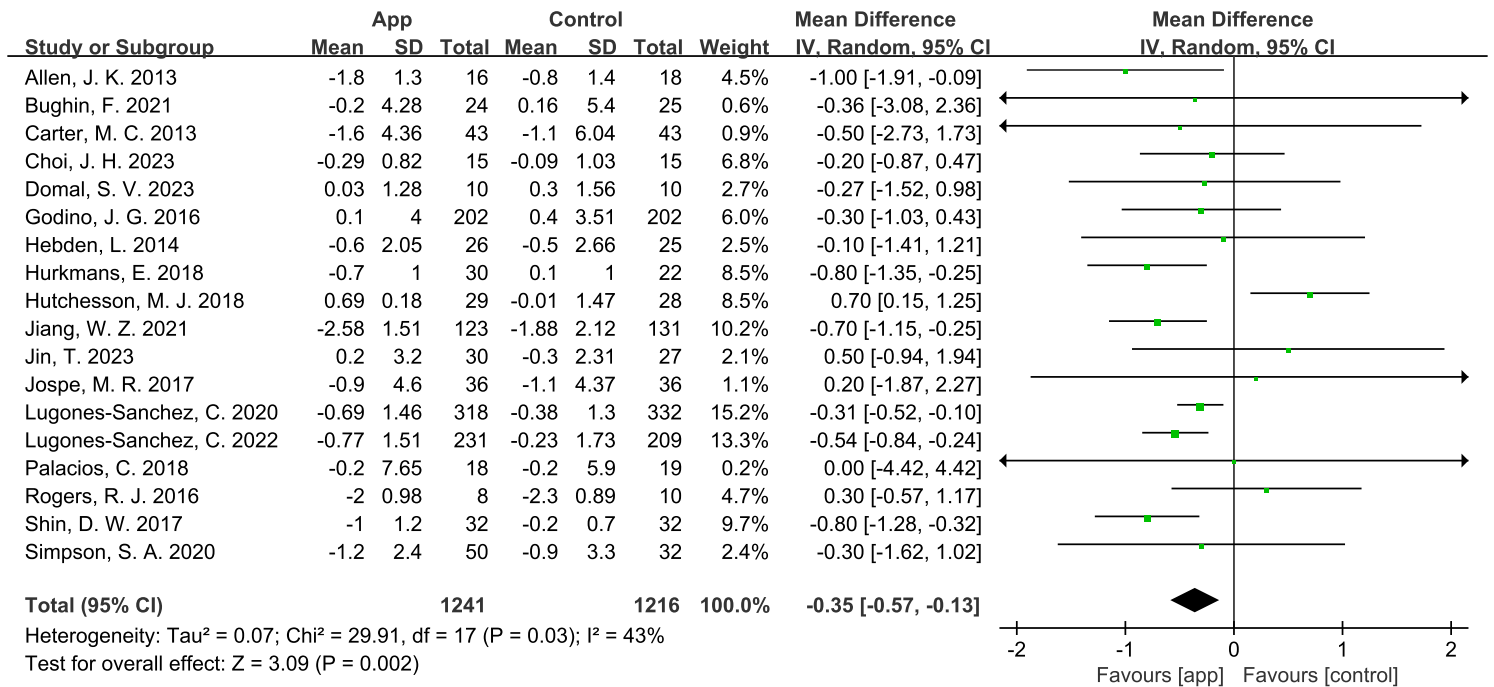

Supplement: Multimedia Appendix 7 [file mhealth-v13-e63313-s007.pdf]

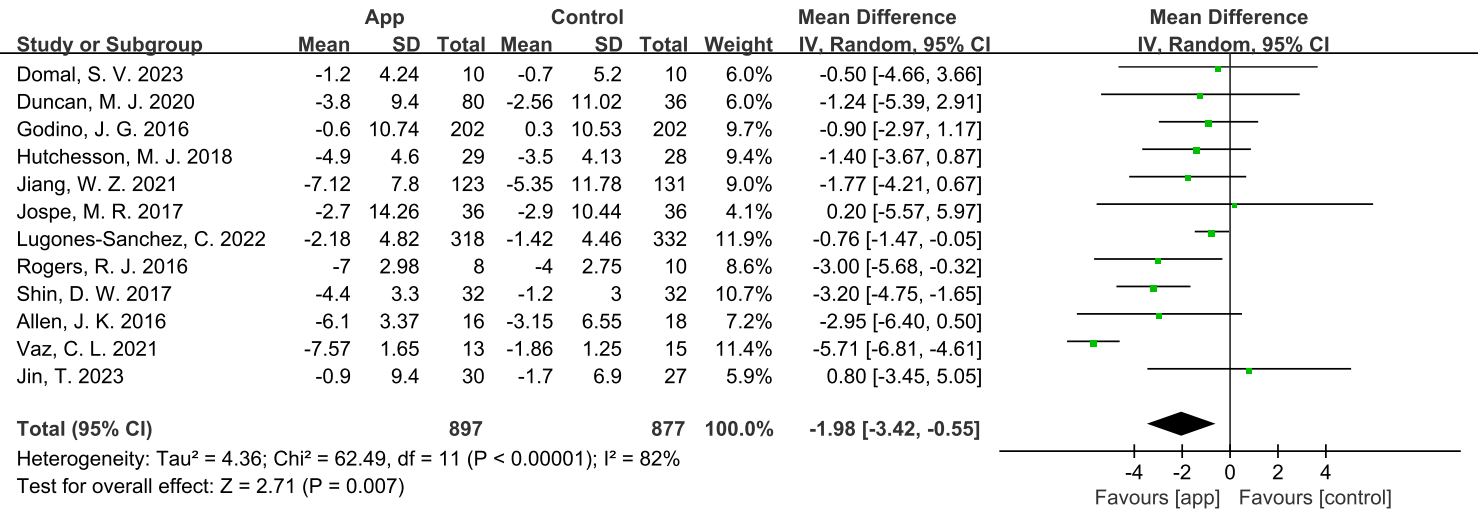

Supplement: Multimedia Appendix 8 [file mhealth-v13-e63313-s008.pdf]

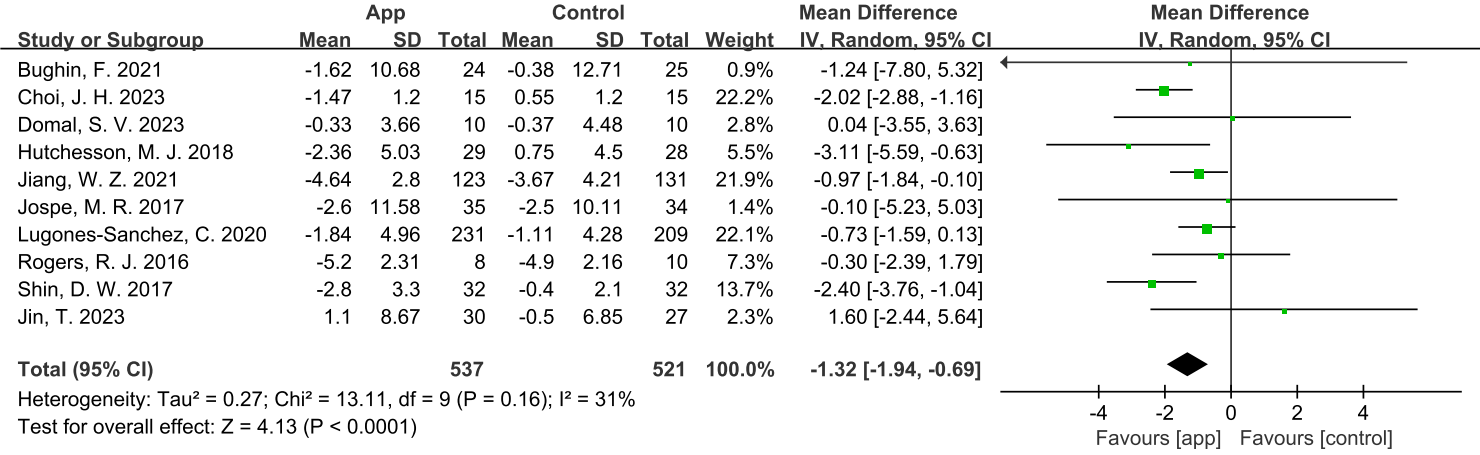

Supplement: Multimedia Appendix 9 [file mhealth-v13-e63313-s009.pdf]

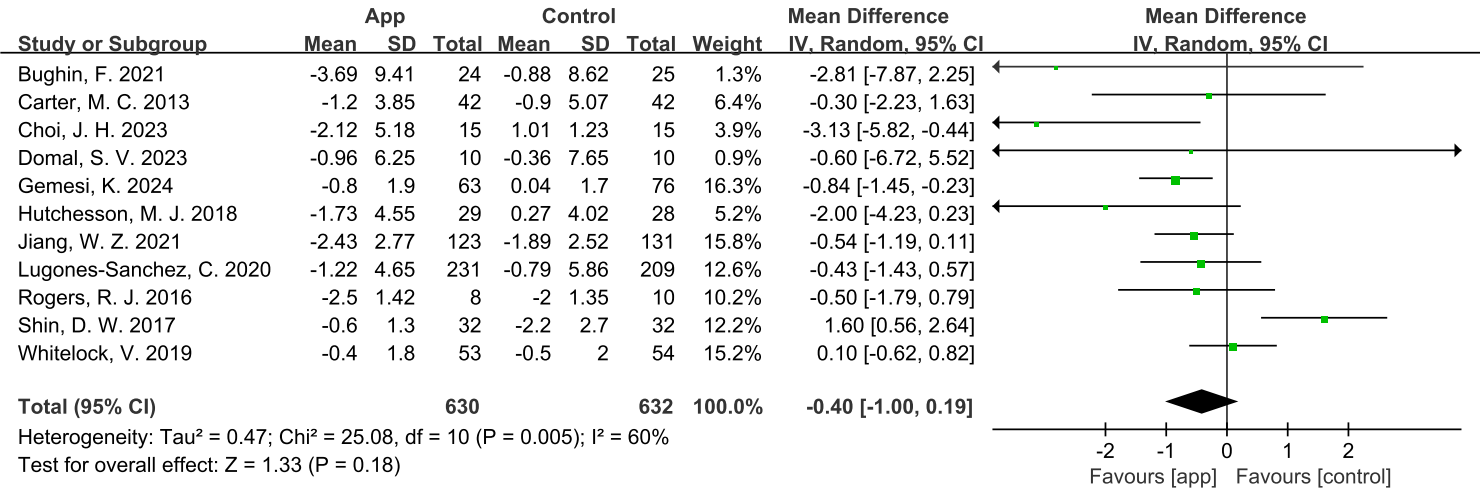

Supplement: Multimedia Appendix 10 [file mhealth-v13-e63313-s010.pdf]

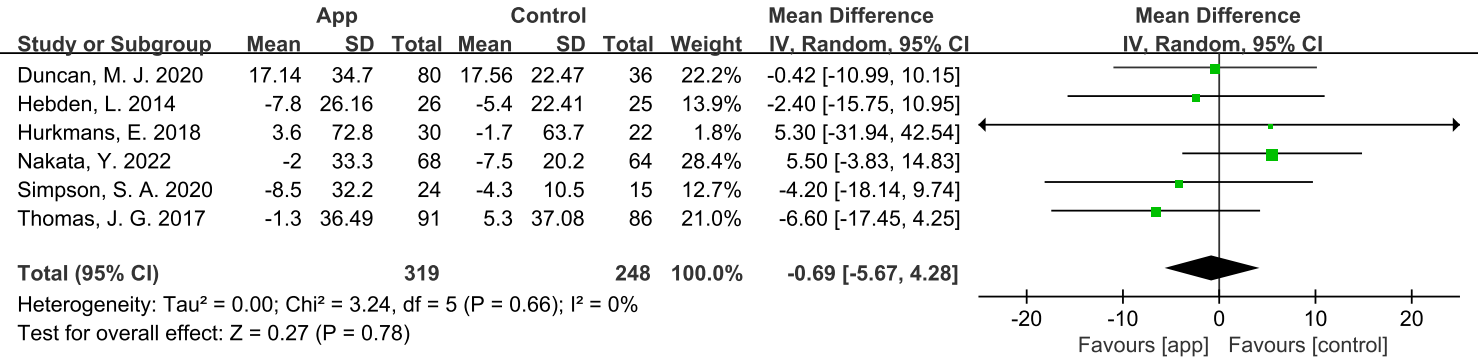

Supplement: Multimedia Appendix 11 [file mhealth-v13-e63313-s011.pdf]

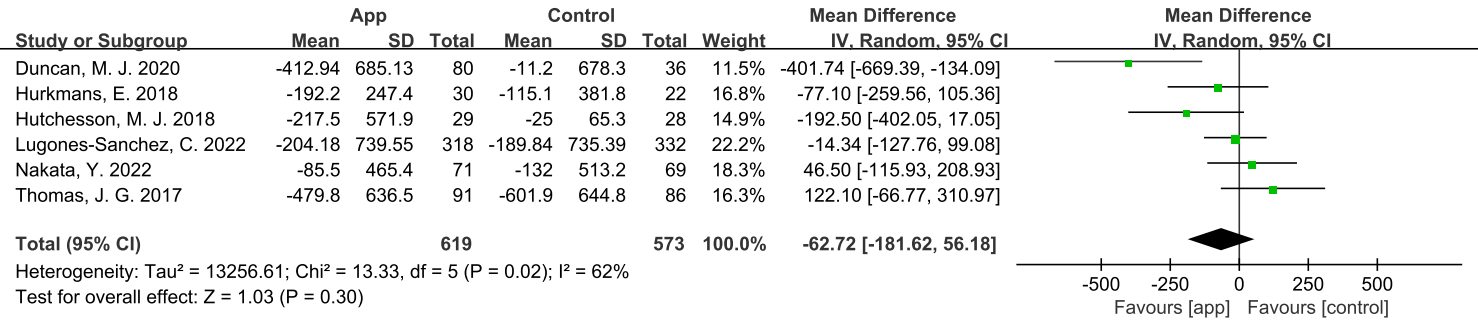

Supplement: Multimedia Appendix 12 [file mhealth-v13-e63313-s012.pdf]

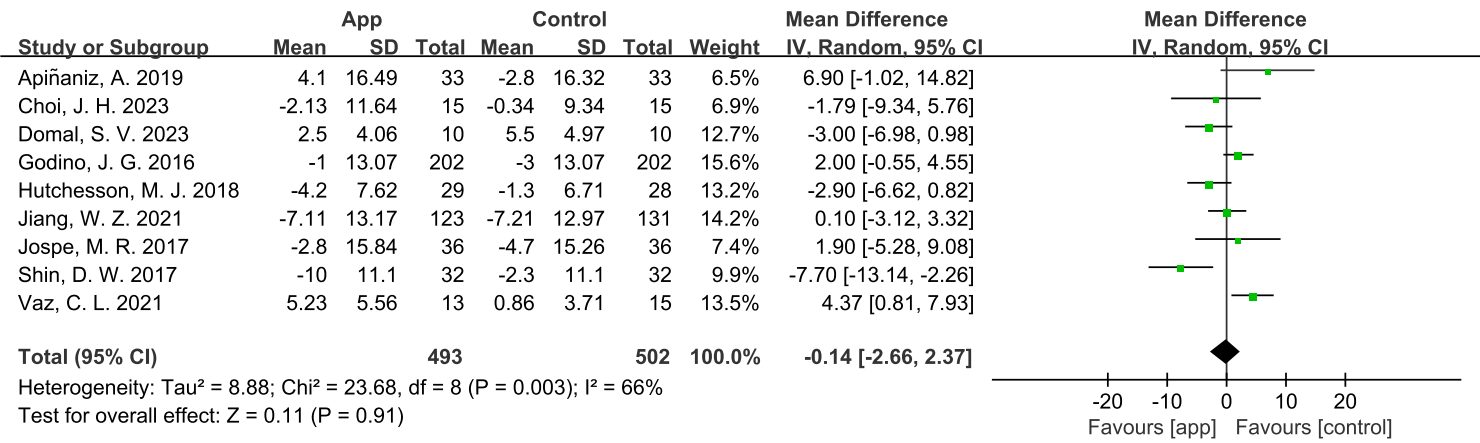

Supplement: Multimedia Appendix 13 [file mhealth-v13-e63313-s013.pdf]

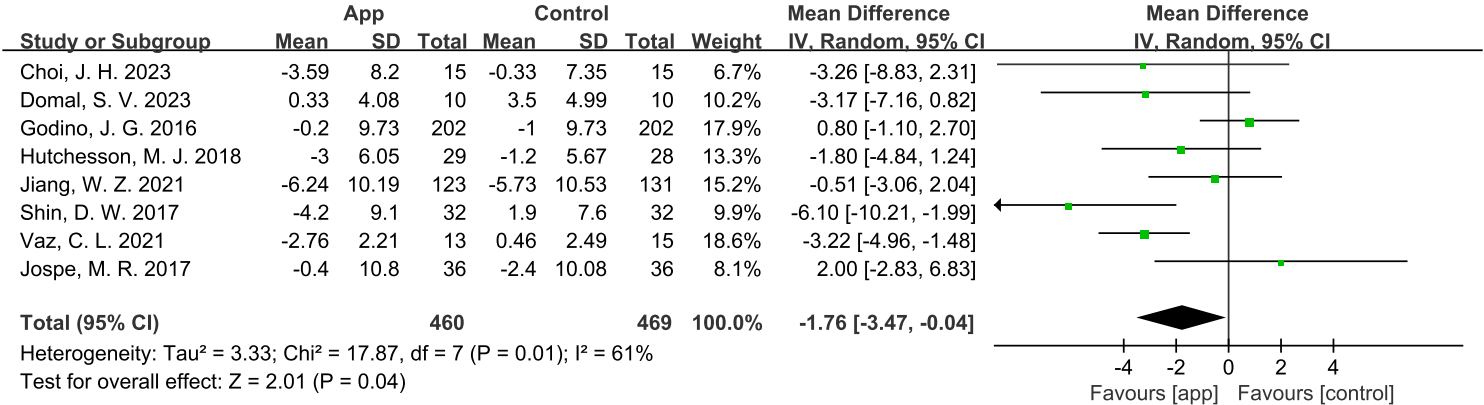

Supplement: Multimedia Appendix 14 [file mhealth-v13-e63313-s014.pdf]

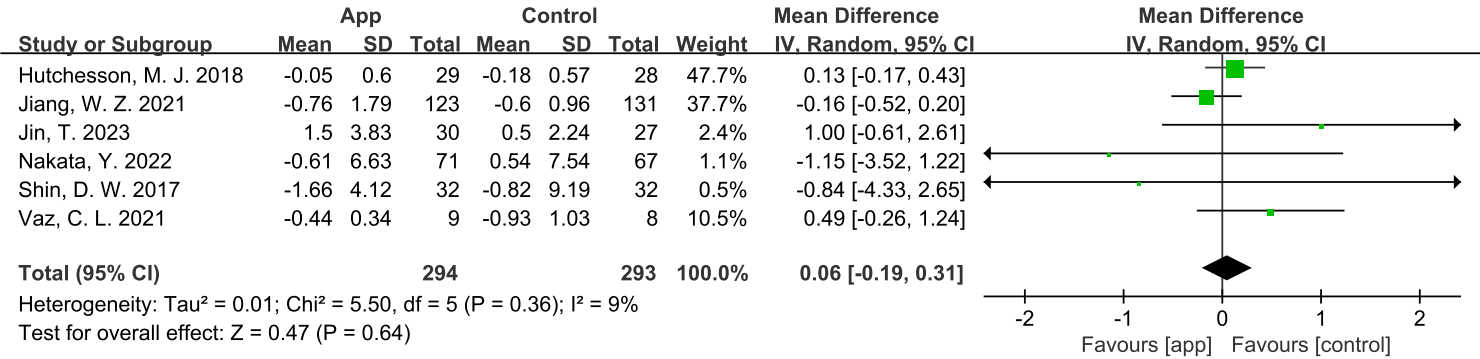

Supplement: Multimedia Appendix 15 [file mhealth-v13-e63313-s015.pdf]

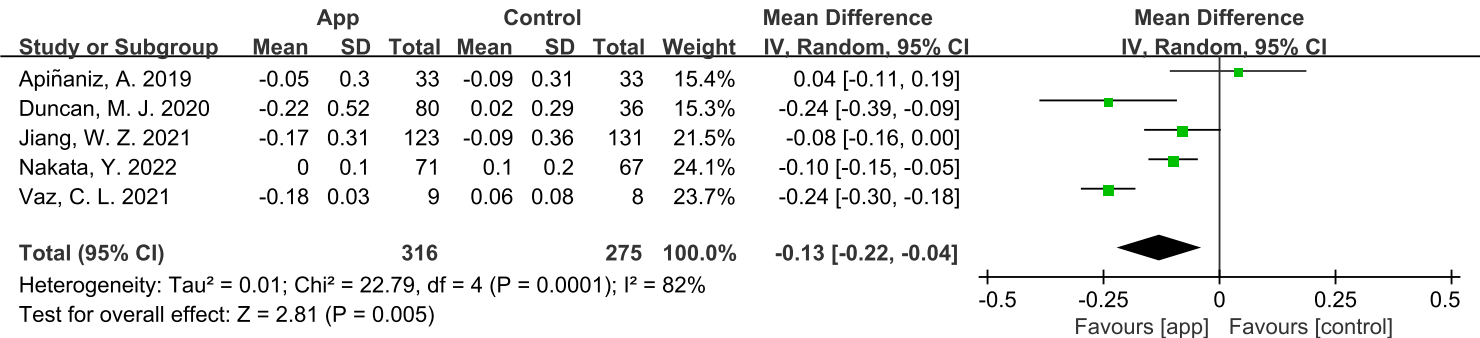

Supplement: Multimedia Appendix 16 [file mhealth-v13-e63313-s016.pdf]

Funnel plot with pseudo 95% confidence limits

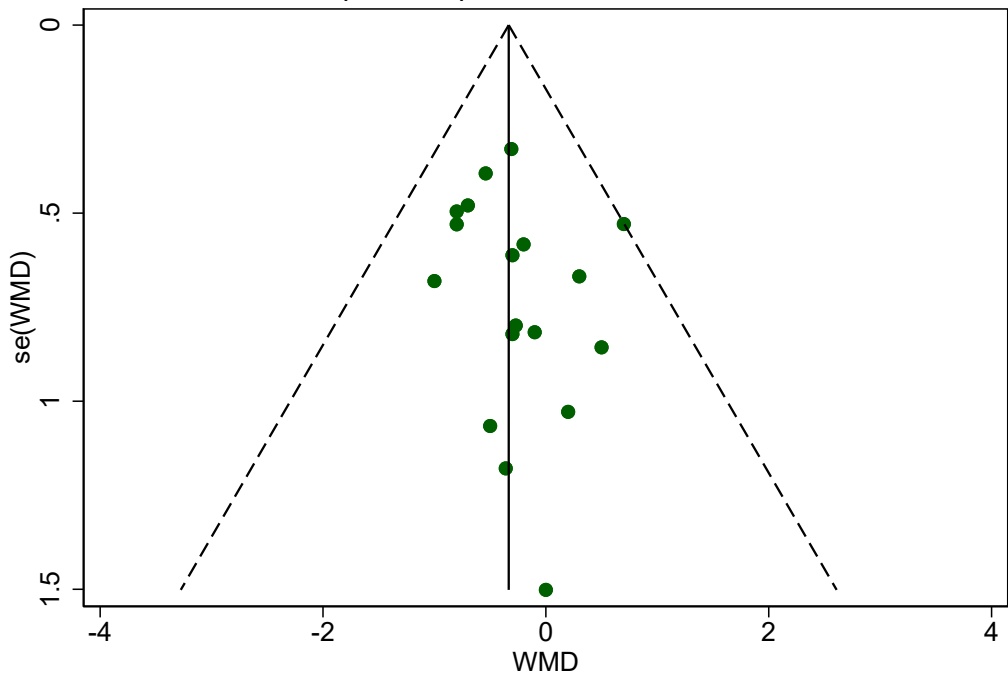

Supplement: Multimedia Appendix 17 [file mhealth-v13-e63313-s017.pdf]

Funnel plot with pseudo 95% confidence limits

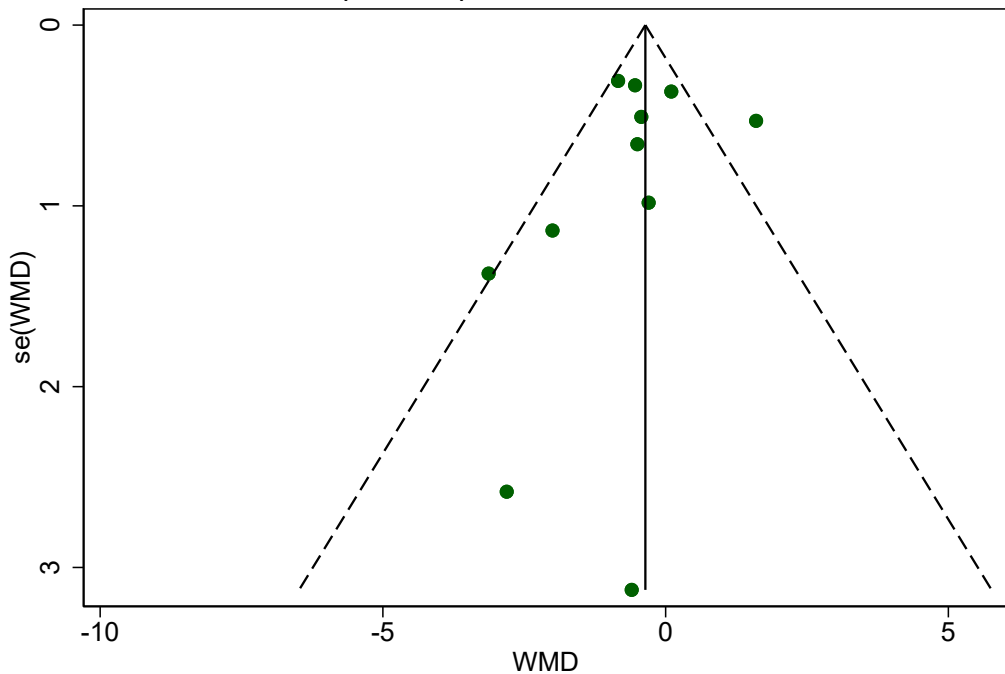

Supplement: Multimedia Appendix 18 [file mhealth-v13-e63313-s018.pdf]

Funnel plot with pseudo 95% confidence limits

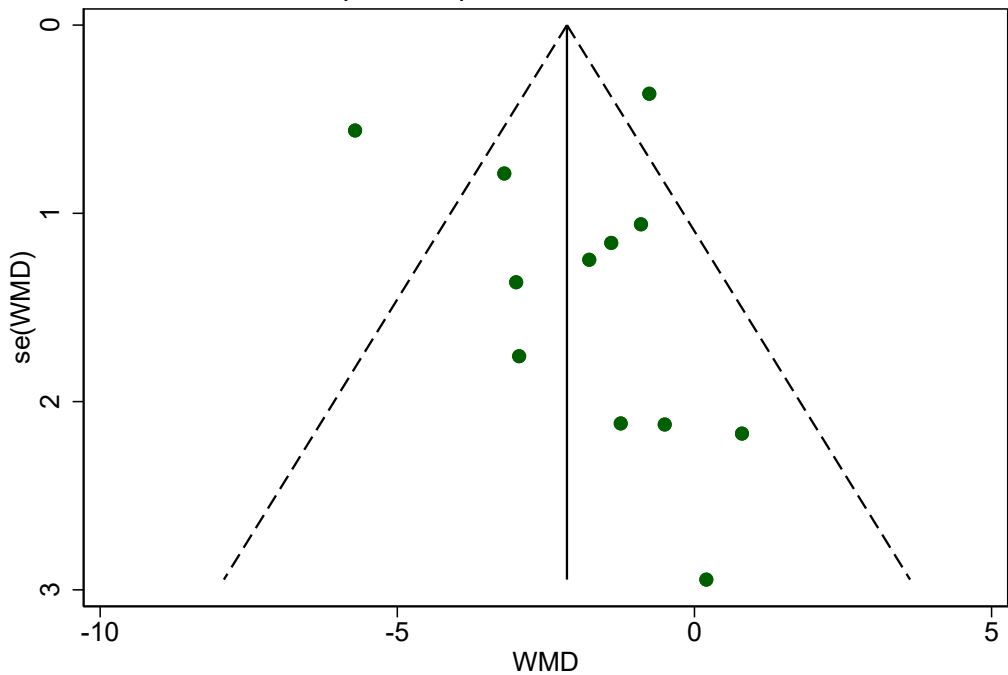

Supplement: Multimedia Appendix 19 [file mhealth-v13-e63313-s019.pdf]

Funnel plot with pseudo 95% confidence limits

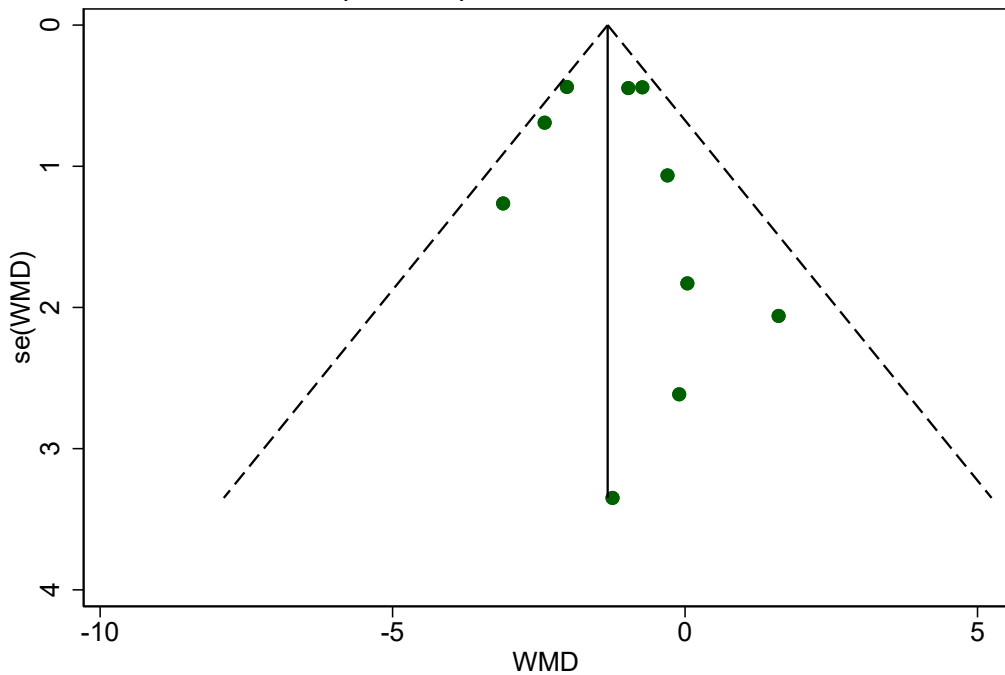

Supplement: Multimedia Appendix 20 [file mhealth-v13-e63313-s020.pdf]
